# Assessing Heavy Metals in the Sele River Estuary: An Overview of Pollution Indices in Southern Italy

**DOI:** 10.3390/toxics12010038

**Published:** 2024-01-03

**Authors:** Fabiana Di Duca, Paolo Montuori, Elvira De Rosa, Bruna De Simone, Immacolata Russo, Raffaele Nubi, Maria Triassi

**Affiliations:** Department of Public Health, University “Federico II”, Via Sergio Pansini 5, 80131 Naples, Italyraf.nubi@gmail.com (R.N.);

**Keywords:** heavy metals, Sele River, contaminant load, water pollution, sediment status, ecological risk assessment, human health risk, non-carcinogenic, carcinogenic

## Abstract

Rapid industrialization, coupled with a historical lack of understanding in toxicology, has led in an increase in estuary pollution, frequently resulting in unexpected environmental situations. Therefore, the occurrence of heavy metals (HMs) constitutes a major environmental issue, posing a serious risk both to aquatic ecosystems and public health. This study aimed to evaluate the levels of eight HMs (As, Hg, Cd, Cr, Cu, Ni, Pb, and Zn) in water, suspended particles, and sediment near the Sele River estuary (Italy) in order to assess their environmental impacts on the sea and health risks for humans. The results revealed an increasing order of HM concentration according to the scheme suspended particulate matter (SPM) > sediment (SED) > dissolved phase (DP) and a moderate contamination status in sediment. The health risk assessment indicated that the non-carcinogenic risk was negligible. Carcinogenic risk, expressed as the incremental lifetime cancer risk (ILCR), was negligible for Cd and Ni and within tolerable limits for As, Pb, and Cr. The findings suggested that, even if there are currently no specific limits for chemical parameters in the transitional waters of Italy, monitoring systems should be implemented to determine pollution levels and implement effective steps to improve river water quality and reduce human health risks.

## 1. Introduction

Human communities have always been clustered around rivers and estuaries for easy access to freshwater and transportation [1]. Moreover, rapid urbanization and industrialization have caused an increase in pollution in coastal areas, especially along the lotic systems that run from rivers and estuaries to offshore coastal regions [2]. Estuaries are positioned at the confluence of land and sea, and they are impacted by both, making their environmental conditions unpredictable [3,4]. The transitional nature of estuaries presents challenges in providing a precise definition, yet they are commonly acknowledged as coastal water bodies strongly affected by tides. Predominantly enclosed by land, estuaries are characterized by a noticeable dilution of seawater caused by freshwater inputs from rivers and runoff [5]. Consequently, the wide array of physical-chemical, climatic, and morphological factors contribute to a significant level of variability. Coupled with pronounced spatial heterogeneity and complexity, each estuarine area emerges as a unique environment with characteristics that defy straightforward generalization and classification [6]. Thus, industrialization has resulted in an increase in the amount of waste dumped in estuaries and river systems, posing issues for both ecosystem and environmental management [7].

Rivers provide a variety of ecological and human services, including water transportation, ecotourism, aquaculture, ecological habitats, and ecological defense [8,9,10]. However, they have been continuously threatened by various contaminants, among which some of the world’s most hazardous contaminants found in riverine aquatic environments are heavy metals (HMs) [11]. In fact, globally, water bodies like rivers and estuaries are considered as potential reservoirs for pollutants, including HMs [12]. Therefore, HM contamination in water bodies is a major environmental problem owing to long-term detrimental impacts on soil-water-air-plant ecosystems and public health [13,14]. 

HMs pose a serious environmental threat to living organisms and aquatic ecosystems due to their non-biodegradability, bioaccumulation, environmental stability, persistence, and biotoxicity characteristics [15]. Furthermore, trace metals can also be delivered and absorbed into the sediments of water bodies by different pathways, which cause long-term adverse effects on living species [16,17]. Thus, HMs can directly affect the physical and chemical properties of sediment and water, inhibiting microbial activities after release from a source [18]. The sediments in estuarine and coastal ecosystems are commonly known to be major sinks for HMs, providing a huge pool for heavy metal storage, but also because when environmental conditions change, these sediments can also be potential sources of HMs for various aquatic organisms, becoming a serious threat to natatory ecosystems [19,20].The rapid increase in the levels of HMs has created a hazard risk of biomagnification of these contaminants, including mercury (Hg), copper (Cu), lead (Pb), cadmium (Cd), chromium (Cr), zinc (Zn), and arsenic (As), through the entrance of noxious elements in the food chain, having acute and chronic impacts on the human body, such as developmental retardation or behavior disorders, kidney damage, abortion, and cancer [3,21,22]. Therefore, it is urgent to investigate the status of HM pollution in water and sediment in order to assess the ecological risks and evaluate their potential sources and fates in dynamic coastal regions [23,24].

The Mediterranean Sea, due to its vulnerable nature and also because of its densely populated coasts and highly developed tourism, is included among water basins for monitoring sources, levels, and effects of persistent toxic pollutants in the environment [25,26,27]. The Mediterranean Sea is recognized as a particularly vulnerable and potentially threatened ecosystem due to anthropogenic inputs. This vulnerability is likely a result of the intense anthropogenic pressure it experiences, compounded by its geographical configuration and location. Being relatively shallow, semi-enclosed, and with limited natural water exchange, the Mediterranean Sea tends to accumulate contaminant inputs rather than disperse them [28,29,30]. In addition, even if in prolific reality rich arable fields are processed using the best farming techniques, several industrial activities in the Sele River plain can generate high loads of chemicals, including HMs, thus having negative effects on the ecosystem and causing health problems and environmental deterioration [31,32,33]. 

This research measured the levels and loads of eight HMs (As, Hg, Cd, Cr, Cu, Ni, Pb, and Zn) in water and sediment samples at and near the Sele River estuary to assess the environmental impact on the Central Mediterranean Sea and health risks for humans caused by exposure to HMs. To date, there are very few studies related to HM impact assessment in this study area, and particularly, no previous research evaluated the HM amounts in the water dissolved phase, suspended particulate matter, and sediment of the Sele River and its input into the Mediterranean Sea. Furthermore, no previous study carried out a human health risk assessment in the study area. The main objectives of the study were (1) to assess the levels in water and sediment and, therefore, the pollution degree caused by HMs in the Sele River; (2) to investigate the spatio-temporal changes in concentrations of HMs; (3) to estimate their inputs into the Mediterranean Sea from the river; 4) to assess the ecological risk characterization by HMs in the Mediterranean Sea area; and 5) to evaluate the human health risks, in terms of non-carcinogenic and carcinogenic risks, associated with exposure by ingestion and dermal contact to HMs in water and sediment from the study area. 

## 2. Materials and Methods

### 2.1. Study Area 

**Figure 1:**
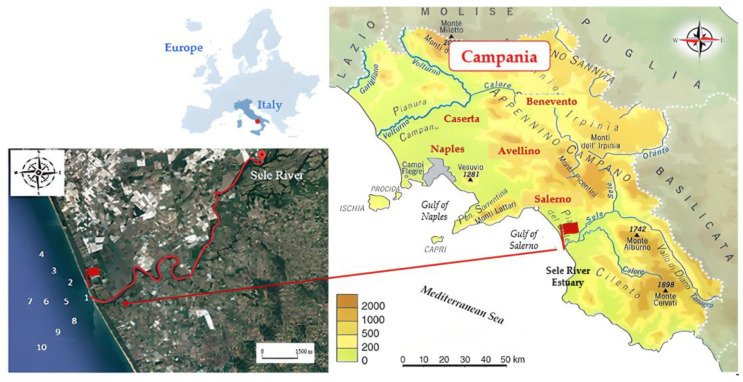
Map of the study area and sampling locations in the Sele River estuary, southern Italy.

The Sele River is located in the Campania Region, in southern Italy (Figure 1). With a length of 64 km, it is the second most important river in the region in terms of average water volume, with a drainage basin of 3235 km^2^ and an annual mean flow rate of 69 m^3^/s [31,32]. The river rises in the province of Avellino near the Caposele, and receives different tributaries, including the Tanagro, the Calore and the Lucano. Then, the river enters the floodplain known as the Sele River plain [34]. 

### 2.2. Sampling

Water samples were collected in four different seasons to assess the spatio-temporal trends in HM concentrations near the Sele River estuary. The sampling was carried out during 2020–2021 in summer (July), autumn (November), winter (February), and spring (April) [33,35,36]. For sediment, samples were collected only in one season (April). The sampling locations are indicated in Figure 1 and the characteristics of each sampling site are detailed in Table 1. Samples were collected at 10 stations, from the river mouth to different distances from it and, in detail, north, west, and south from the estuary. Three water samples aliquots for each sampling location (500 mL) were collected using polypropylene bottles, transported to the laboratory, refrigerated, and stored at +4 °C until analysis, which was carried out within 6 h of sampling. From each sampling site, three sediment samples, each weighing approximately 5 g, were collected at a depth of 0–5 cm using a Van Veen grab sampler. These samples were placed in polyethylene bags and promptly transported to the laboratory within 1 h of collection. Upon arrival, they were cooled at −20 °C until analysis, which was conducted within 1 day of sampling.

### 2.3. Metal Extraction and Analysis 

The methodology used for preparation and extraction was previously described [35,37]. Briefly, water samples were filtered using GF/F glass fiber filters (47 mm × 0.7 µm; Whatman, Maidstone, UK) in order to assess the levels of HMs in the dissolved phase (DP), while filters were used for suspended particulate matter (SPM). Then, samples were acidified with 1% nitric acid (HNO_3_)/chloridric acid (HCl) mixture (*v*/*v*). For sediment, the method consisted of digesting aliquots of 5 g, prior to which samples were air-dried and homogenized [38,39,40,41]. 

Then, the digestion of both sediment and SPM samples was carried out using HNO_3_/HCl (3:1 *v*/*v*) mixture solution with a microwave digestion system (Milestone 1200). Subsequently, the samples were diluted to 25 mL with deionized water and filtered. The analysis was performed by inductively coupled plasma mass spectrometry (ICP-MS) (Thermo Scientific^TM^ ICAP^TM^ RQ) according to method 6020 B proposed by the US EPA (2014) [42], and the amounts of eight HMs (As, Hg, Cd, Cr, Cu, Ni, Pb, and Zn) were evaluated. The tune solution (Ba, Bi, Ce, Co, In, Li, and U, each 1.00 µg/L; (Thermo Scientific, Bremen, Germany)) was used for optimization of the instrumental operating conditions and the kinetic energy discrimination (KED) mode with helium collision gas was applied. Deionized water (18.2 MΩ), HNO_3_ (65% m/m), and HCl (37% m/m) were used. The concentrations of the studied compounds were estimated by the construction of calibration curves (CertiPUR^®^, Merck, Darmstadt, Germany). To check the method, all samples were analyzed in duplicate and each sample was measured in triplicate by Q-ICP-MS detection. 

### 2.4. Quality Control and Quality Assurance

In order to evaluate quality assurance and quality control, duplicates, method blanks, and common reference materials were used. To reduce the background contamination, all glassware, plastics, and quartz were cleaned in 10% HNO_3_ before being washed in deionized water. Triplicate samples and spiked samples were both used to assess the precision of the instrumental procedures. The limits of detection and quantification (LOD and LOQ, respectively) were evaluated in 3 and 10 times the signal/noise ratio for each analyte, respectively. In detail, for water, the LODs were 0.20 µg/L for As, 0.07 µg/L for Hg, 0.15 µg/L for Cd, 0.19 µg/L for Cr, 0.47 µg/L for Cu, 0.52 µg/L for Ni, 0.47 µg/L for Pb, and 0.65 µg/L for Zn. For sediment, the LODs were 0.32 mg/kg dw for As, 0.21 mg/kg dw for Hg, 0.08 mg/kg dw for Cd, 0.29 mg/kg dw for Cr, 0.74 mg/kg dw for Cu, 0.80 mg/kg dw for Ni, 0.56 mg/kg dw for Pb, and 1.04 mg/kg dw for Zn. The LOQs were 0.67 µg/L for As, 0.23 µg/L for Hg, 0.50 µg/L for Cd, 0.63 µg/L for Cr, 1.57 µg/L for Cu, 1.73 µg/L for Ni, 1.57 µg/L for Pb, and 2.17 µg/L for Zn for water. The LOQs for sediment were 1.07 mg/Kg dw for As, 0.70 mg/kg dw for Hg, 0.25 mg/kg dw for Cd, 0.97 mg/kg dw for Cr, 2.47 mg/kg dw for Cu, 2.67 mg/kg dw for Ni, 1.87 mg/kg dw for Pb, and 3.47 mg/kg dw for Zn. For quantification, five-point calibration curves were prepared for each analyte (R^2^ > 0.990). The quality control guidelines of the US EPA were closely followed during each analysis. Routine instrument calibrations were conducted for every analyzed batch or more frequently as needed. Periodic checks were conducted on the method detection limits. For each batch of analysis, reagent blanks, duplicate samples, and sample spike recoveries were all examined for every 10 samples. Certified criteria were established for spiked recoveries (80–120%) and the relative percent difference of duplicate samples (10%). Heavy metal recoveries in spiked samples were in the range of 88–113%, which complied with quality control standards. 

For sediment samples, certified reference material (SD-MEDPOL-1/TM of the International Laboratory of Marine Radioactivity, IAEA, Monaco, Germany) was run after each batch of samples to test the accuracy and precision. Recoveries obtained for sediment-certified reference material samples were in the range of 90–105%.

### 2.5. Statistical Analysis and Heavy Metal Input

Data reported by the study were analyzed using STATA MP v14.0 statistical software (College Station, TX, USA). Recognizing pollution origins and allocating their contribution play an essential role in heavy metal contamination analysis. Techniques like principal component analysis (PCA) are employed to pinpoint sources of heavy metal pollution [35]. PCA, a linear method aimed at reducing complexity, transforms various environmental factors into distinct sources utilizing only a handful of principal components. Its application yields significant insights that encapsulate the entire dataset without compromising the original information. Typically, PCA is employed to uncover relationships among heavy metal variables, aiding in identifying the sources within water surfaces and sediment. Principal component analysis (PCA) was executed. The requirements were previously described [35]. Briefly, the first components with eigenvalues > 1 were considered for the amount of explained variance, and the components had to explain the 70–80% of the variance. A *p*-value < 0.05 was considered to be the significance level. For lower LOQ levels, an LOQ half-value was used. The flow-averaged mean concentration (C_aw_) and annual contaminant loads (F_annual_) were obtained according to UNEP guidelines [43], based on Equations (1) and (2), respectively:

(1) $$F_{annual}=C_{aw}Q_{T}$$

(2) $$C_{aw}=\frac{\sum_{i=1}^{n}C_{i}Q_{i}}{\sum_{i=1}^{n}Q_{i}}$$

where Q_T_ represents the total river input for the study period, assessed using the sum of the monthly averaged water flow; C_i_ is the instantaneous amount; and Q_i_ is the load, expressed as the daily water flow average in kg/year [33,35]. In detail, the cumulative loads of heavy metals (HMs) introduced into the Mediterranean Sea from the Sele River were computed by taking into account the concentration values of individual contaminants at the river mouth during the four months of sampling. Subsequently, the average of the total concentrations was multiplied by the annual mean flow rate (m^3^/year) of the Sele River.

### 2.6. Heavy Metal Pollution Indices in Sediment

The contamination level of HMs in sediment from the Sele River estuary was assessed using the geoaccumulation index (I_geo_), the contamination factor (CF), the pollution index (PLI), and the potential ecological risk index (PERI). 

I_geo_ was used to estimate the contamination status of the environment by comparison against geochemical background concentrations, which was evaluated according to Equation (3) [35,44]:

(3) $$I_{geo}={Log}_{2}\left[\frac{C_{n}}{{1.5\;B}_{n}}\right]$$

where C_n_ and B_n_ are the amount detected in the sediment sample and the geochemical background concentration for an individual HM (n), respectively. Specifically, the background values considered for each heavy metal under investigation were: 5.00 mg/kg dw (As), 0.07 mg/kg dw (Hg), 0.05 mg/kg dw (Cd), 90.00 mg/kg dw (Cr), 45.00 mg/kg dw (Cu), 68.00 mg/kg dw (Ni), 20.00 mg/kg dw (Pb), and 95.00 mg/kg dw (Zn) [45,46]. A factor of 1.5 indicated the background matrix correlation value, which represented the possible variation due to the lithogenic effect and weathering [47]. As a qualitative scale of pollution intensity, I_geo_ includes seven classes on the basis of the degree of contamination. Therefore, based on the I_geo_ value, a site can be classified from unpolluted to extremely polluted, as showed in Table 2. 

Moreover, the status of HM contamination in sediment was further assessed using the contamination factor (CF), contamination degree (CD), and pollution load index (PLI), which resulted in the cumulative indication of the overall level of heavy metal pollution in sediment from the research area. Therefore, the CF, CD and PLI indices were evaluated using Equations (4)–(6) below [44,48,49,50]:

(4) $$CF=\frac{C_{n}}{B_{n}}$$

(5) $$CD=\sum_{i=1}^{n}{CF}_{i}$$

(6) $$PLI=\sqrt[{n}]{{CF}_{1}\times {CF}_{2}\times \ldots \times {CF}_{n}}$$

where n represents the number of HMs evaluated. 

The potential ecological risk index (PERI), applied to estimate the potential ecological risk of the studied HMs in sediment samples collected in the study area, allows an ecological risk assessment to be carried out by combining ecological and environmental effects with toxicological data, and it was calculated using Equations (7) and (8) [35,51]:

(7) $$E_{r}^{i}={T_{r}}^{i}\times CF$$

(8) $$PERI=\sum_{i=1}^{n}E_{r}^{i}$$

where T_r_^i^ indicates the toxic response factor of individual HMs, as shown in Table 3.

### 2.7. Ecological Risk Index for Sediment

The Sediment Quality Guidelines (SQGs) were used to estimate the potential risk for aquatic organisms and water systems associated with HMs in sediment [53]. In detail, threshold effect level (TEL), probable effect level (PEL), effect range low (ERL), and effect range median (ERM) values were considered [54,55,56]. These values are indicated in Table 4. Therefore, when amounts were lower than the ERL and TEL values, no adverse effects would be observed. In addition, HM concentrations were considered rarely to be toxic for values below the ERL and above the TEL. In contrast, when levels were above the ERM and PEL values, adverse effects on the ecosystem could be predicted [50,57].

### 2.8. National Recommended Water Quality Criteria

The toxicity of HMs in water samples from the Sele River estuary was assessed according to the National Recommended Water Quality Criteria by the US EPA [58]. Therefore, when the HM amount in the water phase exceeds its corresponding criterion continuous concentration (CCC) value, chronic toxicity may occur in the aquatic ecosystem. In contrast, acute toxicity may occur when the HM concentration detected in the water phase exceeds its corresponding criterion maximum concentration (CMC) value [59].

### 2.9. Health Risk Assessment: Carcinogenic and Non-Carcinogenic Risks

A health risk assessment estimates the probability of any given amount of adverse health effects occurring during a certain time period [60,61]. A contaminant’s health risk is generally based on an estimation of the risk level and is categorized as carcinogenic or non-carcinogenic health risk [62,63].

Therefore, the chronic daily intakes (CDIs) for ingestion (CDI_ingestion_) and dermal (CDI_dermal_) exposure were evaluated according Equations (9) and (10), proposed by the U.S. Environmental Protection Agency (US EPA) [64].

(9) $${CDI}_{ingestion}=\frac{C_{w}\times DAI\times ABS\times EF\times EP}{BW\times AT}$$

(10) $${CDI}_{ingestion}=\frac{C_{w}\times DAI\times ABS\times EF\times EP}{BW\times AT}$$

According to Mohammadi et al., Table 5 shows the parameters used for risk assessment [61].

Also, the hazard quotient (HQ) and hazard index (HI) were used to assess the potential non-carcinogenic health risk induced by heavy metal levels in water via ingestion and dermal absorption [61]. The USEPA proposed guidelines for assessing the non-carcinogenic and carcinogenic risks via ingestion and dermal absorption pathways [64,67]. Therefore, the evaluation was conducted according to Equations (10) and (11). To estimate the HQ, the reference oral doses (RfDs) through oral ingestion and dermal absorption routes were used, as indicated in Table 6. When HQ > 1, adverse non-carcinogenic effects on health may occur, while for HQ values below 1, no risk is predicted [60,64].

(11) $$HQ=\frac{CDI}{RfD}\;\;$$

The HI, expressed as the sum of individual HQs, is used to evaluate the total non-carcinogenic risk due to exposure to a mixture of potentially toxic chemicals in water systems [70,71]. When HI > 1, non-carcinogenic dangers are predicted; however, HI < 1 denotes that exposed people are unlikely to have obvious negative health effects [60,61].

(12) $$HI=\sum_{k=1}^{n}HQ={HQ}_{As}+{HQ}_{Hg}+{HQ}_{Cd}+{HQ}_{Cr}+{HQ}_{Cu}+{HQ}_{Ni}+{HQ}_{Pb}+{HQ}_{Zn}\;$$

Furthermore, the incremental lifetime cancer risk (ILCR) was used to assess the probable cancer risks caused by total exposure (ingestion and dermal contact) to HM concentrations in water. It was estimated according to Equation (12).

(13) ILCR=CDI×CSF

where CSF represents the cancer slope factor (CSF), expressed as kg/day/mg, which correlates with the average exposure concentration leading to an increase in the probability of developing cancer. The CSFs applied in this study are displayed in Table 7.

## 3. Results

### 3.1. Levels of Heavy Metals in the Water Dissolved Phase (DP)

The concentrations of HMs found in the dissolved phase (DP) ranged from nd to 14.28 μg/L (with a mean value of 1.74 ± 2.88 μg/L) for As, from nd to 2.35 μg/L (with an average of 0.27 ± 0.53 μg/L) for Hg, from nd to 1.69 μg/L (average of 0.28 ± 0.43 μg/L) for Cd, from nd to 14.97 μg/L (average of 2.63 ± 3.77 μg/L) for Cr, from nd to 13.23 μg/L (mean value of 1.34 ± 2.56 μg/L) for Cu, from nd to 11.15 μg/L (average value of 1.56 ± 2.53 μg/L) for Ni, from nd to 14.41 μg/L (mean value of 0.88 ± 2.50 μg/L) for Pb, and from nd to 5.46 μg/L (average of 1.02 ± 1.45 μg/L) for Zn. The results obtained for water dissolved phase (DP) samples collected at the sampling stations near the river during the research period (2020–2021) are shown in Table S1.

### 3.2. Levels of Heavy Metals in the Suspended Particulate Matter (SPM)

The concentrations of HMs detected in the suspended particulate matter (SPM) samples collected from the sampling locations near the Sele River estuary ranged from nd to 116.52 μg/L (with a mean value of 17.77 ± 24.82 μg/L) for As, from nd to 10.03 μg/L (with an average of 1.77 ± 2.00 μg/L) for Hg, from nd to 4.57 μg/L (average of 0.50 ± 0.86 μg/L) for Cd, from nd to 33.47 μg/L (average of 4.94 ± 6.28 μg/L) for Cr, from nd to 139.85 μg/L (mean value of 25.36 ± 30.99 μg/L) for Cu, from nd to 88.74 μg/L (average value of 11.81 ± 17.53 μg/L) for Ni, from nd to 86.66 μg/L (with a mean value of 19.44 ± 21.72 μg/L) for Pb, and from nd to 107.54 μg/L (average value of 14.45 ± 24.20 μg/L) for Zn. The results found for SPM at 10 sampling locations near the Sele River estuary during four seasons (2020–2021) are displayed in Table S2.

### 3.3. Levels of Heavy Metals in the Sediment

The levels of individual and total HMs detected in sediment samples collected at 10 sampling locations near the Sele River estuary during four seasons (2020–2021) are illustrated in Table S3. Regarding individual HMs, the levels found were in the range of nd to 12.56 mg/kg dw(with a mean value of 3.68 ± 3.60 mg/kg dw) for As and nd–1.10 mg/kg dw(with a mean value of 0.27 ± 0.44 mg/kg dw) for Hg. Moreover, the concentrations ranged from nd to 0.98 mg/kg dw (mean value of 0.19 ± 0.40 mg/kg dw), from 1.22 to 29.10 mg/kg dw (mean value of 9.22 ± 8.84 mg/kg dw), and from nd to 7.32 mg/kg dw (mean value of 2.22 ± 2.34 mg/kg dw) for Cd, Cr, and Cu, respectively. Additionally, the mean value detected for Ni was 4.26 ± 7.80 mg/kg dw (range of nd–24.89 mg/kg dw) and 6.72 ± 10.31 mg/kg dw (range of nd –34.57 mg/kg dw) for Pb. Finally, the levels found for Zn were in the range of 5.92–42.00 mg/kg dw (with a mean value of 17.62 ± 12.29 mg/kg dw).

### 3.4. Heavy Metal Inputs and Spatio-Temporal Distribution

The total annual HM load from the Sele River estuary into the Tyrrhenian Sea (central Mediterranean Sea) was 483.97 kg/year. Specifically, regarding the individual contribution of each HM, the load was 81.69 kg/year for As, 8.98 kg/year for Hg, 3.37 kg/year for Cd, 32.95 kg/year for Cr, 111.02 kg/year for Cu, 60.25 kg/year for Ni, 112.81 kg/year for Pb, and 72.90 kg/year for Zn.

**Figure 2:**
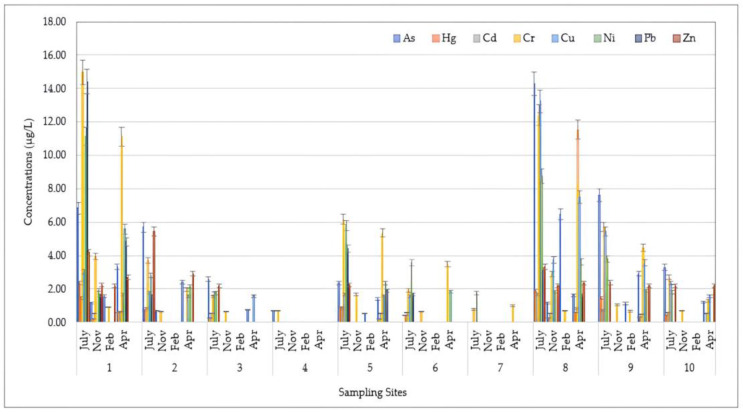
Spatio-temporal distribution of heavy metals (HMs) detected in the water dissolved phase (DP) at 10 sampling locations (Site 1: Mouth; Site 2: River Mouth 500 m North; Site 3: River Mouth 1000 m North; Site 4: River Mouth 1500 m North; Site 5: River Mouth 500 m West; Site 6: River Mouth 1000 m West; Site 7: River Mouth 1500 m West; Site 8: River Mouth 500 m South; Site 9: River Mouth 1000 m South; Site 10: River Mouth 1500 m South) from the Sele River estuary, in southern Italy, during four sampling campaigns (2020–2021).

**Figure 3:**
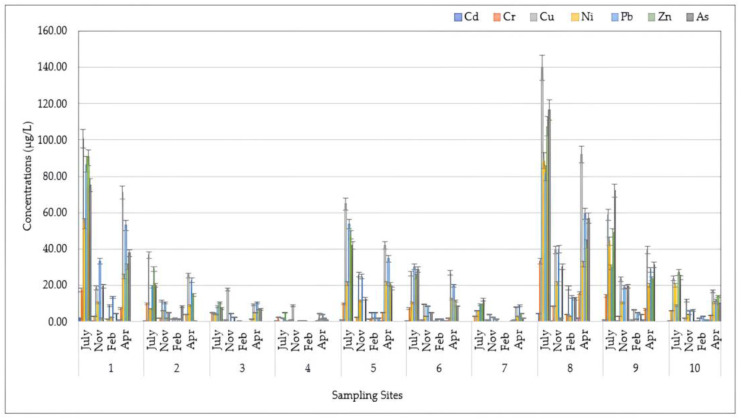
Spatio-temporal distribution of heavy metals (HMs) detected in suspended particulate matter (SPM) at 10 sampling locations (Site 1: Mouth; Site 2: River Mouth 500 m North; Site 3: River Mouth 1000 m North; Site 4: River Mouth 1500 m North; Site 5: River Mouth 500 m West; Site 6: River Mouth 1000 m West; Site 7: River Mouth 1500 m West; Site 8: River Mouth 500 m South; Site 9: River Mouth 1000 m South; Site 10: River Mouth 1500 m South) from the Sele River estuary, in southern Italy, during four sampling campaigns (2020–2021).

**Figure 4:**
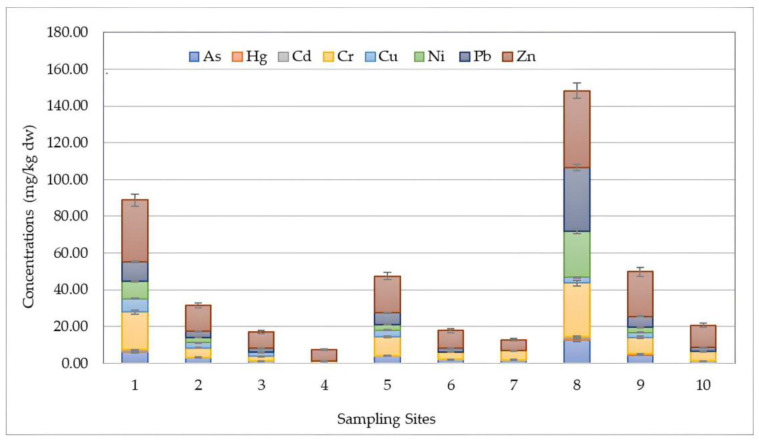
Spatial distribution of heavy metals (HMs) detected in sediment (SED) samples collected at 10 sampling locations (Site 1: Mouth; Site 2: River Mouth 500 m North; Site 3: River Mouth 1000 m North; Site 4: River Mouth 1500 m North; Site 5: River Mouth 500 m West; Site 6: River Mouth 1000 m West; Site 7: River Mouth 1500 m West; Site 8: River Mouth 500 m South; Site 9: River Mouth 1000 m South; Site 10: River Mouth 1500 m South) from the Sele River estuary, in southern Italy.

The spatial distributions of HMs for DP, SPM, and SED samples assessed at different sampling locations during four seasons are shown in Figure 2, Figure 3 and Figure 4, respectively.

Figure 2 displays the spatio-temporal distribution of HMs detected in DP samples collected during the research period. Higher levels were detected at the mouth and 500 m to the south (500S) during the warmer and drier seasons (July > April). In detail, the highest concentrations were recorded at the mouth for Hg (2.35 μg/L), Cr (14.97 μg/L), Ni (11.15 μg/L), and Pb (14.41 μg/L) in the dry season (July). For As and Cu, the highest amounts were found in the same period, with maximum values of 14.28 μg/L and 13.23 μg/L, respectively. In contrast, lower levels of HMs were detected during the winter season (February < November). Moreover, the results showed that the HM pollution degree (expressed as the total concentration) was greater at the mouth (with an average amount over four seasons of 26.28 ± 23.99 μg/L) and 500S (with an average amount over four seasons of 27.02 ± 23.17 μg/L), which decreased considerably in the northern direction, with a minimum value recorded at the 1500N location (0.34 ± 0.68 μg/L).

Figure 3 illustrates the spatio-temporal distribution of HMs in SPM samples from the Sele River estuary. A quick glance reveals that a higher amount was found at 500S (500 m to the south) during the hot season (July), with a maximum value of 582.64 μg/L (53,948.15 mg/kg dw), expressed as the total amount recorded. Regarding individual analytes, the highest concentrations detected during this sampling period at the 500S site were 116.52 μg/L for As, 10.03 μg/L for Hg, 4.57 μg/L Cd, 33.47 μg/L for Cr, 139.85 μg/L for Cu, 88.74 μg/L for Ni, and 107.54 μg/L for Zn. Only for Pb was the greatest amount recorded at the mouth (86.66 μg/L). Therefore, a similar pattern to that observed for DP was also observed in this instance, with a higher degree of HM pollution found in the summer season (July) and near the mouth (500S), and with a decreasing trend moving in the northern direction. Particularly, regarding spatial distribution, the highest total level of HMs was found at 500S (271.49 ± 231.94 μg/L, expressed as the average value over four seasons).

Figure 4 shows the spatio-temporal distribution of HMs in sediment samples near the Sele River estuary. Similar to the DP and SPM phases, the highest concentration in the sediment phase, amounting to 147.65 mg/kg dw, expressed as the total amount recorded, was also detected at 500S (500 m to the south) during the sampling season (April), followed by the mouth (88.19 mg/kg dw). Considering the amounts detected for each analyte, the highest concentrations were found at the 500S location for As (12.56 mg/kg dw), Hg (1.10 mg/kg dw), Cr (29.10 mg/kg dw), Ni (24.89 mg/kg dw), Pb (34.57 mg/kg dw), and Zn (42.00 mg/kg dw). However, for Cd and Cu, the highest levels were obtained at the mouth, corresponding to values of 0.28 and 7.32 mg/kg dw, respectively. Therefore, an increasing trend moving in the southern direction was also observed for sediment.

**Figure 5:**
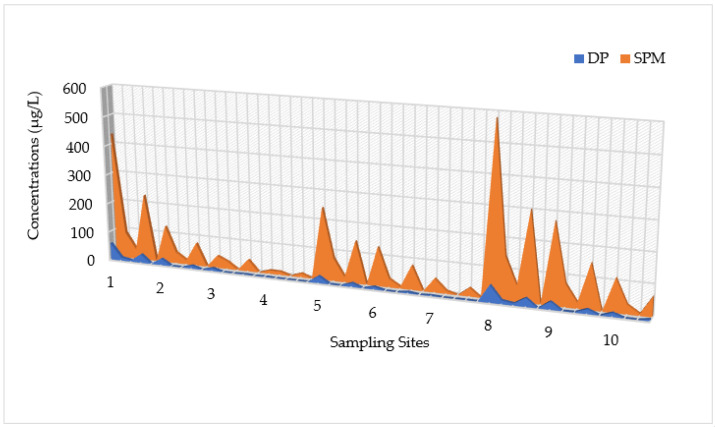
Spatial distributions of heavy metal (HM) concentrations (µg/L) compared for dissolved phase (DP) and suspended particulate matter (SPM) samples collected in the study area at 10 sampling locations (Site 1: Mouth; Site 2: River Mouth 500 m North; Site 3: River Mouth 1000 m North; Site 4: River Mouth 1500 m North; Site 5: River Mouth 500 m West; Site 6: River Mouth 1000 m West; Site 7: River Mouth 1500 m West; Site 8: River Mouth 500 m South; Site 9: River Mouth 1000 m South; Site 10: River Mouth 1500 m South).

**Figure 6:**
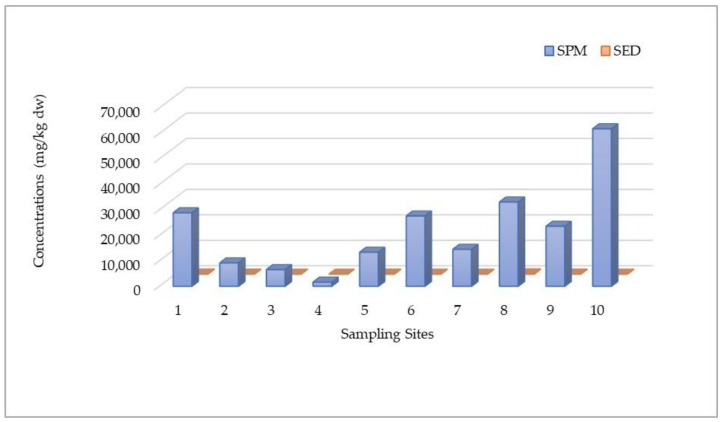
Spatial distributions of heavy metal (HM) concentrations (mg/kg dw) compared for SPM and SED samples collected in the study area at 10 sampling locations (Site 1: Mouth; Site 2: River Mouth 500 m North; Site 3: River Mouth 1000 m North; Site 4: River Mouth 1500 m North; Site 5: River Mouth 500 m West; Site 6: River Mouth 1000 m West; Site 7: River Mouth 1500 m West; Site 8: River Mouth 500 m South; Site 9: River Mouth 1000 m South; Site 10: River Mouth 1500 m South).

Figure 5 illustrates the comparison between HM amounts detected in the DP and SPM phases. The figure suggests that higher levels were found in SPM samples. In addition, the HM concentration values found in the SPM phase were significantly higher than those found in the sediment phase, expressed as the total amount recorded in the same period of collection (April) (Figure 6). In fact, in contrast to the much lower concentration found in sediment (147.65 mg/kg dw), the highest total amount was found in SPM (33,102.17 65 mg/kg dw), recorded at the same site (500S) and during the same sampling period (April). Accordingly, the partition coefficients (K_p_), assessed as the ratio between HM amounts detected in the SED and SPM samples, were evaluated. Also, the ratios of HM concentrations obtained in the SPM samples and those in the DP samples were calculated. Particularly, the results of K_p_ indicated an increasing trend in HM amount from DP to SED and more to SPM. Therefore, the findings revealed an increasing order of HM concentration according the scheme SPM > SED > DP.

Data were also reported as the sum of DP + SPM. Specifically, the total HM concentrations found in the water matrix (as the sum of both phases) were in the range of nd to 641.29 µg/L, with a mean value of 105.78 ± 138.47 µg/L (evaluated for all sampling campaigns and sites). Moreover, the highest amount was found at location 8 (mean value of 298.51 ± 255.05 µg/L over four seasons). Moreover, the total HM levels found in the water matrix (sum of DP and SPM) for each analyte in detail were in the range of nd to 130.80 µg/L (mean value of 19.53 ± 27.26 µg/L) for As, nd–11.90 µg/L (with an average of 2.03 ± 2.50 µg/L) for Hg, nd–6.26 µg/L (mean value of 0.78 ± 1.25 µg/L) for Cd, nd–45.88 µg/L (mean value of 7.58 ± 9.67 µg/L) for Cr, nd–153.08 µg/L (average of 26.70 ± 33.20 µg/L) for Cu, nd–97.47 µg/L (mean value of 13.37 ± 19.75 µg/L) for Ni, nd–101.07 µg/L (mean value of 20.32 ± 23.67 µg/L) for Pb, and nd–110.86 µg/L (mean value of 15.48 ± 25.20 µg/L) for Zn.

Compared to previous studies, the HM levels found in DP samples collected around the Sele River estuary were lower than those evaluated near the Sarno River estuary and Tiber River estuary by Montuori et al., who reported that total HM concentrations were in range of 0.32–1680.39 μg/L and 11.80–1359.67 μg/L, respectively [26,72]. Instead, a recent study carried out in an area close to that of this study, near the estuary of the Volturno River, in southern Italy, recorded total HM concentrations similar to those obtained in this study (range of 0.20–65.67 μg/L) [35]. For the suspended phase, again, the amounts reported by Montuori et al. for the Sarno and Tiber River estuaries were higher (ranges of 103.6–7734.6 μg/L and 18.74–2841.56 μg/L, respectively) [26,72] than those found in this study, although they were comparable to those detected by De Rosa et al. near the Volturno River estuary (1.37–601.77 μg/L) [35]. Compared to previous research conducted in southern Italy on HM amounts in sediment samples, the results obtained in this study showed lower values than those obtained for sediments collected near the estuaries of the Sarno and Tiber Rivers (in the range of 90.7–2470.3 mg/Kg dw and 42.8–1686.8 mg/kg dw, respectively), but similar to those obtained around the Volturno River estuary (14.62–157.33 mg/kg dw) [26,35,72].

### 3.5. Statistical Analysis

For statistical analysis, PCA was employed for the three phases (dissolved phase, suspended particulate matter, and sediment). Particularly, the findings of the study indicated that the HM contamination level was higher for locations sampled south of the mouth. Furthermore, a similar situation was observed for the sediment (SED) and dissolved phase (DP) data, indicating that only the greatest eigenvalue was greater than 1, and the first component contributed to 83.78% and 80.15% of the total variance, respectively (Figures S1 and S2). Furthermore, all variables contributed to the determination of the first principal component with comparable impact, indicating that the first component was an average of each metal (variable), since the presence of all studied HMs was observed at each site. For suspended particulate matter (SPM), in addition to the eight studied HMs, the weight of filter variable was considered. The results showed that this variable was orthogonal to the others, which were high correlated with one another, so there were both the components that explained the total variance of the variables (HMs) and the component that explained the variance of the weight of filter variable. The two highest eigenvalues were larger than 1 and the two first components explained 96.15% of the total variance (Figure S3).

### 3.6. Heavy Metal Contamination Status

**Figure 7:**
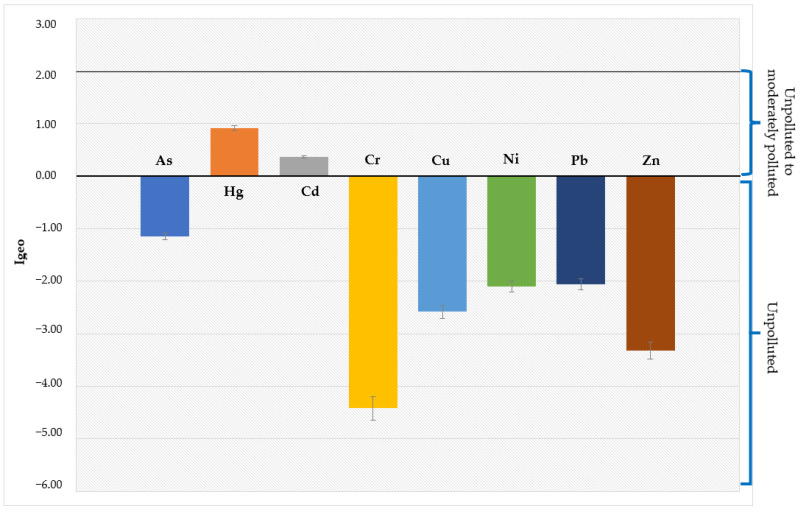
Geoaccumulation index (I_geo_) values for individual heavy metals (HMs) in sediment (SED).

**Figure 8:**
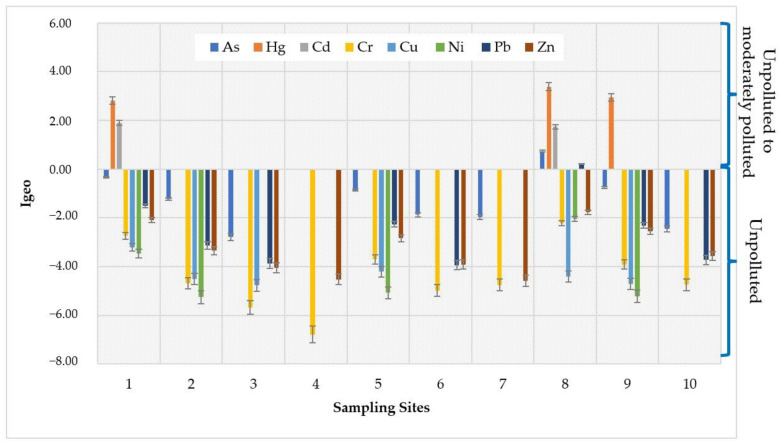
Geoaccumulation index (I_geo_) values for individual heavy metals (HMs) estimated at 10 sampling locations near the Sele River estuary, in southern Italy.

The contamination status caused by HMs in the study area was estimated using I_geo_. Table 8 and Figure 7 and Figure 8 show the I_geo_ values evaluated in SED samples from the Sele River estuary, in southern Italy. Particularly, higher values of I_geo_ max were found for As and Hg at site 8 (500S) and for Cd at site 1 (the mouth). On the other hand, lower values were recorded for Cr and Ni at locations 4 (1500N) and 2 (500N), respectively.

Moreover, in order to evaluate the status of HM contamination in sediment collected in the study area, the CF, CD, PLI, and PERI indices were estimated. The results are indicated in Table 9.

**Figure 9:**
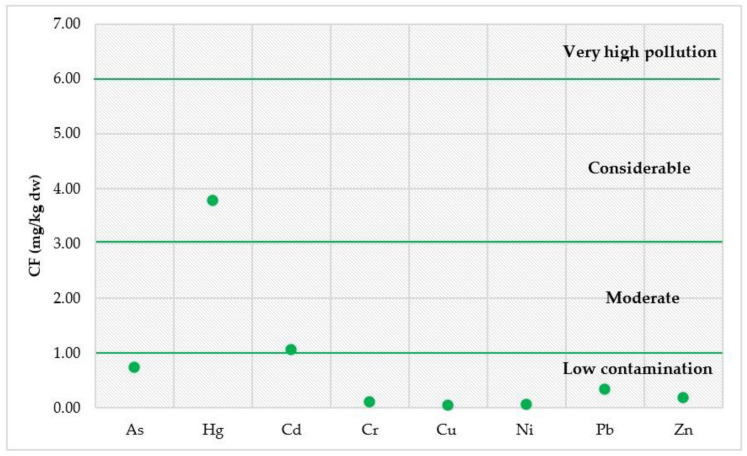
Contamination factors (CFs) evaluated for sediment (SED) samples collected during the sampling campaign near the Sele River estuary.

Regarding the CF, the results obtained are displayed in Figure 9. The highest CF values were found for Hg and Cd, with mean values of 3.79 and 1.06, respectively.

### 3.7. Ecological Risk Assessment

**Figure 10:**
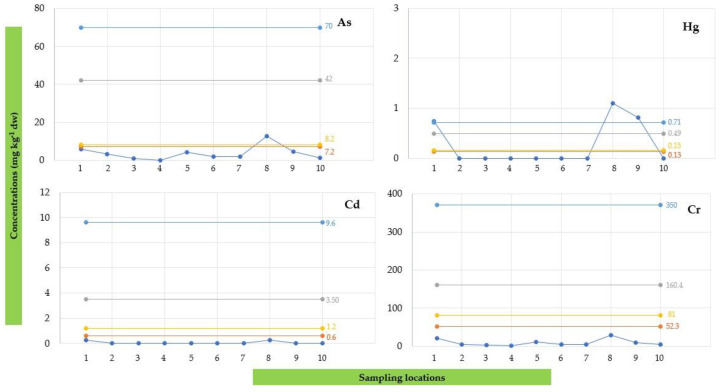
Comparison of the concentrations obtained in the sediment (SED) samples for As, Hg, Cd, and Cr with threshold effect level (TEL, orange line), probable effect level (PEL, grey line), effect range low (ERL, yellow line), and effect range median (ERM, blue line).

**Figure 11:**
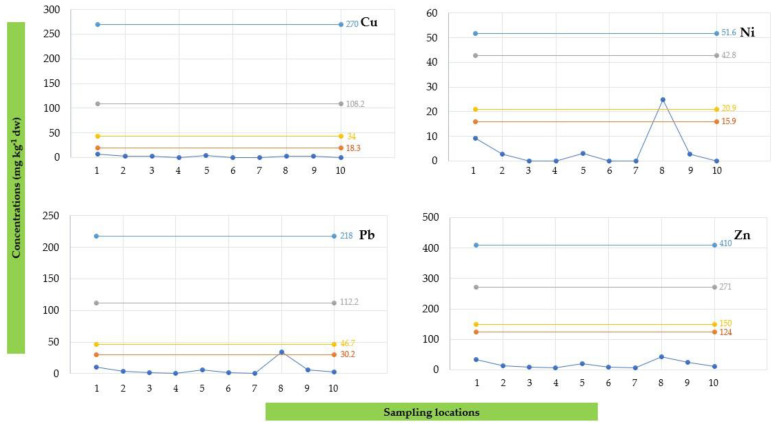
Comparison of the concentrations obtained in the sediment (SED) samples for Cu, Ni, Pb, and Zn with threshold effect level (TEL, orange line), probable effect level (PEL, grey line), effect range low (ERL, yellow line), and effect range median (ERM, blue line).

The potential ecological risk for aquatic organisms and the water system associated with the presence of HMs in sediment was estimated by comparing the concentrations detected in samples collected from the Sele River estuary with the SQGs. Therefore, the comparisons with TEL, PEL, ERL, and ERM values are displayed in Figure 10 for As, Hg, Cd, and Cr, and in Figure 11 for Cu, Ni, Pb, and Zn.

### 3.8. National Recommended Water Quality Criteria

Table 10 shows the comparison of HM amounts detected in DP samples collected in the study area during four seasons with the National Recommended Water Quality Criteria proposed by the US EPA [58].

### 3.9. Health Risk Assessment

**Figure 12:**
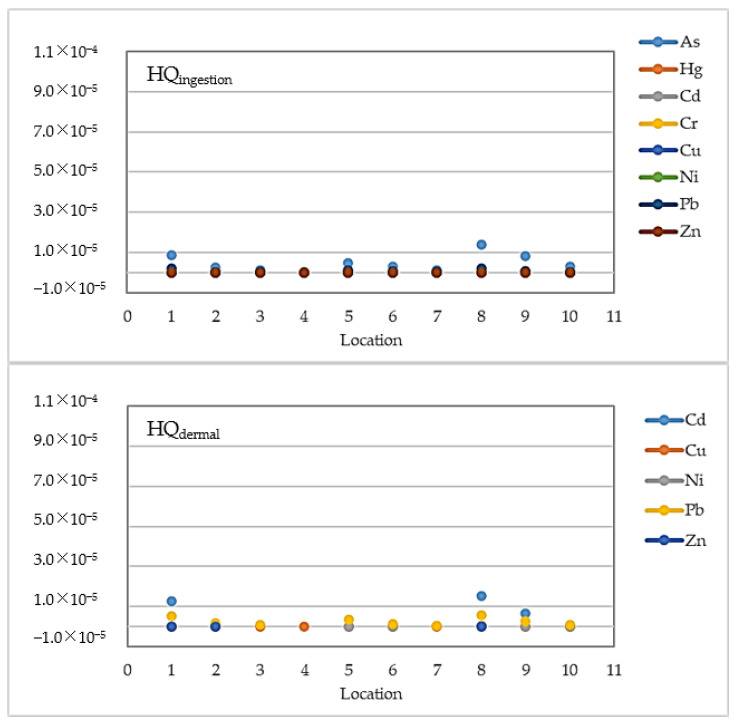
Graphical representation of the hazard quotients (HQs) obtained in this study.

In order to estimate the potential non-cancer health risk due to ingestion and dermal absorption of HMs in water sampled near the Sele River estuary, the CDIs, HQs, and HIs were evaluated. The results obtained are indicated in Table 11 and Table 12 and Figure 12. Particularly, in Table 11, minimum, maximum, and mean levels of CDIs for adults via ingestion and dermal contact routes of HMs in water samples from the study area are shown.

As given in Table 11, the mean levels of CDI_dermal_ were higher than those of CDI_ingestion_, with the highest mean values recorded for Zn (9.49 × 10^−6^ mg/kg/day) > Cu (1.77 × 10^−6^ mg/kg/day) > Pb (1.38 × 10^−6^ mg/kg/day) > As (1.29 × 10^−6^ mg/kg/day). These values were used for HQs. The highest risks were estimated to be from As (1.98 × 10^−6^) through ingestion and from Cr (5.69 × 10^−5^) by dermal contact.

Figure 12 illustrates the graphical representation of the HQs evaluated in this study for eight HMs (As, Hg, Cd, Cr, Cu, Ni, Pb, and Zn).

Moreover, the minimum, maximum, and mean values of HIs evaluated in this study for HM exposure through different pathways (ingestion and dermal contact) are shown in Table 12.

The carcinogenic risk assessment is summarized in Table 13, which indicates the incremental lifetime cancer risk (ILCR) values obtained for total exposure caused by ingestion and dermal contact in the study area.

## 4. Discussion

The study evaluated the impact of heavy metals in the Mediterranean Sea from the Sele River, in southern Italy. Therefore, HM amounts were estimated in water (as DP and SPM) and sediment samples collected near the estuary of the river. Moreover, since only a few studies have evaluated HMs in both phases, it is difficult to compare the results obtained in this study with those of other studies. Table S4 shows a comparison of heavy metal concentrations in previous studies carried out from other river catchments and transitional waters in areas close to the study area. Therefore, compared to a few previous studies, the HM concentrations found in the water samples collected near the Sele River estuary (as the sum of DP and SPM) were lower than those detected in other Italian rivers [26,72], but comparable with those found in samples collected near the Volturno River estuary [35].

Higher amounts of HMs were observed at the river’s mouth and at 500 m moving southward, possibly reflecting that HM pollution downstream is more serious than that upstream, which is typically less influenced by human activity [24]. Regarding individual HMs, Cr showed the highest levels in DP, probably because Cr is not easily absorbed by suspended particles, resulting in restricted Cr input in sediment. As a result, Cr may be found mostly in bodies of water [73]. In contrast, Cu was detected in the highest amount in SPM and Zn showed the highest level in the sediment phase. Accordingly, Cu and Zn, consistently found in environmentally significant amounts in sediments worldwide, mainly originate from vehicle emissions and atmospheric deposition [74]. However, they may be easily accumulated in river sediment, which is the ultimate sink for HM pollution [75]. Thus, these findings could be due to the fact that they are mostly collected in sediment through adsorption, sedimentation, or flocculation. Although these activities may significantly decrease the HM levels in the overlying water body, the same HMs chelated by sediments can be released into the water through the same mechanisms under specific environmental circumstances, resulting in secondary contamination [74,76]. To date, there are various strategies to mitigate the presence of HMs in river water and maintain low concentrations. Accordingly, the implementation of best management practices (BMPs) in industrial and agricultural activities can significantly reduce the runoff of pollutants into rivers. Additionally, public awareness campaigns and regulatory measures play pivotal roles in encouraging responsible waste disposal practices and fostering a collective commitment to preserving water quality. Employing a combination of these approaches can contribute to the sustainable management of heavy metal levels in river water. The results of this study suggest that monitoring programs are required for assessing pollution levels and establishing effective measures to enhance river water quality and decrease human health threats.

The spatio-temporal distributions of HMs in DP and SPM samples collected during four seasons in 2020–2021 at 10 locations near the estuary were evaluated. For both phases, the highest levels were detected at the mouth and 500 m to the south (500S) during the warmer and drier seasons (July and April). For sediment, samples were collected only in one season (April) and the spatial distribution indicated higher levels detected 500 m to the south, followed by the mouth. The data obtained suggested that fresh inputs of these contaminants to the sea from the Sele River estuary can occur, and the SPM phase could act as carrier from the sediment to the water phase. In fact, due to their chemical characteristics, HMs can bind to the sediment phase but perturbation phenomena such as rain or earth movements can facilitate their transition to the water phase, which occurs through the suspended phase. These findings are in agreement with those of Huang et al. [75], who stated that the bioavailability of Cu and Zn in river sediment may be affected by seasonal variations and spatial distribution [75]. Particularly, they assessed that seasonal variations may influence HM contamination in sediment, mainly in the dry season [75]. Accordingly, recent studies carried out in Ghana and India revealed that concentrations of Cu and Zn in river sediment were higher in the dry season than in the wet season [77,78]. The results of this investigation were also consistent with those by Zhao et al., who observed that seasonal changes in these pollutant levels may be caused by anthropogenic pressures and different climatic conditions. In fact, they investigated the spatio-temporal distribution patterns of HMs in water samples collected in the Yitong River (China) and found higher amounts of HMs during the summer season and in urban areas than in the winter and in suburban areas [79]. On the contrary, Liu et al. stated that levels of HMs found in the surface water of Qingjiang River (China) were all higher during the wet season than in the dry season [80].

The total annual HM load from the Sele River estuary into the Mediterranean Sea was evaluated. Compared with earlier investigations conducted nearby, the total HM load from the Sele River estuary to the sea was lower than that reported by De Rosa et al., who evaluated the annual HM input at about 620.39 kg/year from the Volturno River estuary to the Mediterranean Sea [35], and drastically lower than that reported by Montuori et al. from the Sarno River estuary (13,977.53 kg/year) [72]. However, these findings suggest that the Sele River could be a significant point source of HM discharge into the Mediterranean Sea, even though the concentrations of the individual HMs observed, and therefore their inputs, were low.

The HM contamination status of sediment from the Sele River estuary was assessed using I_geo_, CF, PLI, and PERI. The average I_geo_ values indicated that, based on the classification proposed by Hossain et al. [44], the sampling locations could be considered as unpolluted with regard to As, Cr, Cu, Ni, Pb, and Zn and unpolluted/moderately polluted for Hg and Cd. In contrast, the maximum values of I_geo_ at sites 8 (500S) for Hg and 1 (mouth) for Cd indicated the sites were moderately and heavily polluted, respectively. Moreover, in order to evaluate the status of HM contamination in sediment collected in the study area, the CF, CD, PLI and PERI indices were estimated. According to Khadanga et al., data obtained regarding CF for Hg and Cd suggested considerable and moderate contamination, respectively [50]. For CD, the results indicated a moderate contamination level for the river [48]. In addition, the PLI value indicated baseline levels of HMs in sediment collected from the study area [49]. According to the E_r_ and PERI values evaluated, Hg could entail a high ecological risk for the Sele River, followed by Cd with a considerable ecological risk for the river [51]. Thus, since the mean value of PERI was in the range of ≥150 to < 300, the river could be a source of HMs, mainly Hg and Cd, with considerable/moderate levels of pollution by HMs [51]. Effectively, Hg, released both from natural and anthropogenic sources, mostly enters aquatic environments by riverine input. Moreover, the literature states that industrial sewage discharge and watershed and atmospheric deposition are major sources of Hg in the sediments of rivers [81,82]. In addition, the development of industry and agriculture has led to a significant increase in Cd levels in sediment because of its extensive application in fertilizer and pesticide production [83].

Concerning the ecological risk for aquatic organisms and water systems associated with the presence of HMs in sediment, the amounts recorded in sediment samples collected from the Sele River estuary were compared with the SQGs. Particularly, the amount of As detected at 500S (12.56 mg/kg dw) was higher than the TEL and ERL values (Figure 11). Moreover, the amounts of Hg detected at the mouth (0.74 mg/kg dw), at 500S (1.10 mg/kg dw), and 1000S (0.81 mg/kg dw) were higher than the TEL, PEL, ERL, and ERM values. For Ni, the amount found at 500S (24.89 mg/kg dw) was above the TEL and ERL values, while the amount of Pb detected at the same site (34.57 mg/kg dw) was higher than the TEL value. On the other hand, for Cd and Cr (Figure 10) and for Cu and Zn (Figure 11), no concentration value exceeded the SQGs. Therefore, according to the study results, heavy metal concentrations found near the Sele River may suggest an issue of concern. Consequently, continuous monitoring of these types of environmental contaminants is necessary to preserve the aquatic ecology of the river.

Regarding the comparison of HM amounts detected in DP samples collected in the study area during four seasons with the National Recommended Water Quality Criteria proposed by the US EPA [58], the results showed that all of the values found for As and Zn were below the CMCs and CCCs indicated for river and marine waters. On the contrary, Hg had higher concentrations than the CMC and CCC. Specifically, the levels of Hg exceeded the CMC and CCC for freshwater in 60 and 18% of the samples, respectively, and for saltwater in 6 and 19% of the samples, respectively. For Cd, the observed levels were above the CCC for freshwater in 27% of the samples, while the amount of Ni exceeded the CCC for saltwater in 13% of the samples, unlike Cr, for which 58% of the samples were over the CMC for saltwater. Moreover, the Cu amount exceeded the CMC and CCC for saltwater in 10 and 31% of the samples, respectively, while for Pb, the amount surpassed the CCC values for freshwater and saltwater in 14% and 6% of the samples, respectively. Therefore, the findings indicate that there may be a possible risk connected with the heavy metals under consideration.

Furthermore, this research computed the health risks of HMs, some of which exhibit proven toxicity to humans. Dashtizadeh et al. [84] stated that the chronic intake of HMs, such as Cd, Cr, As, and Pb, has considerable biological toxicity and is dangerous to human health. According to this study, Cd mainly accumulates in the human hepatic system and kidneys, disturbing estrogen secretion, and is also classified as carcinogenic. In addition, Cr, Cu, and Zn can cause non-carcinogenic hazards such as neurologic involvement, headache, and liver disease, while acute and chronic arsenic exposure can also cause dermal, respiratory, reproductive and carcinogenic effects [84]. Moreover, Kan et al. reported that Pb exposure can cause gastrointestinal damage, child amentia, and Alzheimer’s disease, while Hg exposure may result in kidney damage, central nervous system defects, arrhythmia, and respiratory problems [85]. For the non-carcinogenic risk assessment, the CDI_dermal_ values showed higher mean levels than those of CDI_ingestion_, with the highest mean values recorded for Zn and Cu. The values of HQ indicated that the highest risks were associated with As due to accidental ingestion and Cr caused by dermal contact. However, the HQs for all examined HMs were lower than the level of concern (HQ <  1). Furthermore, the HIs were evaluated to estimate the total potential non-carcinogenic impact, and all values were below 1 (HI < 1), suggesting that no non-carcinogenic concerns were to be expected [85,86]. Accordingly, the findings of the mean HQs suggested an acceptable level of non-carcinogenic health risk in all samples taken near the Sele River estuary. Therefore, the non-carcinogenic risk from heavy metals via accidental ingestion and dermal contact with water near the Sele River estuary was revealed to be within a safe range for the exposed population. The carcinogenic risk of the studied HMs in the Sele River was estimated as the incremental lifetime cancer risk (ILCR). However, for Hg and Zn, CSF values are not available, and consequently, the carcinogenic risk assessment was not carried out [61,69]. Similarly, Yang et al. used Cd, Pb, and As for both carcinogenic and non-carcinogenic risk assessments, while they included Hg only in the non-carcinogenic assessment [87]. The results indicated that considering the total exposure caused both by ingestion and dermal contact, the carcinogenic risk was negligible for Cd (ILCR _mean_ = 9.28 × 10^−7^), and Ni (ILCR _mean_ = 6.54 × 10^−7^). However, it was within the acceptable range of 10^−6^ to 10^−4^ for As (ILCR _mean_ = 2.94 × 10^−6^), Pb (ILCR _mean_ = 1.78 × 10^−5^), and Cr (ILCR _mean_ = 4.91 × 10^−5^) [87]. The acceptable risk limit is expected to be 10^−6^ in the case of single element carcinogenic risk and 10^−4^ in the event of multi-element carcinogenic risk [88]. Additionally, according to Mohammadi et al. [61], for an individual HM, when the ILCR value is less than 1 × 10^−6^, the cancer risk is considered negligible. On the other hand, an ILCR value higher than 1 × 10^−4^ suggests that the cancer risk is considerable. For the total of all heavy metals through all exposure routes, the acceptable level is 1 × 10^−5^ [81]. Among all of the studied HMs, Cr showed the highest ILCR value (ILCR _max_ = 2.90 × 10^−4^), implying that exposure over a long lifetime via ingestion and dermal contact could increase the probability of cancer to a significant level for exposed people. In contrast, Ni presented the lowest chance of cancer risk.

## 5. Conclusions

This study provides data on the occurrence of eight heavy metals (As, Hg, Cd, Cr, Cu, Ni, Pb, and Zn) in water and sediment, as well as their spatial and seasonal patterns, in the Sele River estuary (Italy). Furthermore, the study offers the first assessment of the health hazards to humans from HM exposure in the study region using non-carcinogenic and carcinogenic analyses. The study’s findings reveal that greater levels of HMs were detected at the river’s mouth and 500 m moving southward, implying that HM pollution downstream is more severe than that upstream, which is normally less impacted by human activities. Furthermore, the spatio-temporal distributions revealed that the greatest amounts were discovered during the warmer seasons (July and April), meaning that fresh inputs of these pollutants into the sea are possible, and the SPM phase might serve as a carrier from the sediment to the water phase. Furthermore, the HM contamination status of sediment from the Sele River estuary was moderate, suggesting that the river could be a source of considerable/moderate levels of pollution by HMs, mainly Hg and Cd. In terms of health risk assessment, the study’s outcomes showed that there was no detectable non-carcinogenic risk for the analyzed trace elements since the HQs for all studied HMs were lower than the thresholds of concern. The carcinogenic risk, expressed as the incremental lifetime cancer risk (ILCR), was negligible for Cd and Ni and within the acceptable range for As, Pb, and Cr. This study significantly advances environmental and scientific research by comprehensively assessing heavy metal levels in Sele River water and sediment. The spatio-temporal analysis provides a detailed perspective on heavy metal concentrations, aiding the understanding of variations over time and space. Notably, it estimates heavy metal inputs from the Sele River into the Mediterranean Sea, emphasizing the interconnectedness of fluvial and marine ecosystems. The study’s characterization of ecological risk in the Mediterranean Sea and assessment of human health risk contribute to a broader understanding of environmental impacts. Its multi-faceted analysis, spanning local and Mediterranean contexts, methodology, and extensive scope, positions it as a crucial reference for future studies on heavy metal contamination and associated risks to ecosystems and human health. To address heavy metal contamination in estuarine environments, key actions involve implementing continuous monitoring systems for timely detection of variations, regulating industrial and urban discharges, and engaging in ecosystem regeneration projects like wetland creation and reforestation. Community involvement is vital, requiring awareness initiatives for sustainable practices. Comprehensive environmental impact studies are essential before new activities are undertaken near estuaries to prevent negative effects on water and sediment quality. Controlling terrestrial pollution sources, fostering international collaboration for shared estuarine regions, and implementing preventive measures are crucial steps to address heavy metal pollution on a transboundary level.

## Figures and Tables

**Table 1:** Sampling locations with identification number (ID), characteristics of each site, coordinates, and abbreviation.

Sampling Locations				
ID	Characteristics	Latitude (N)	Longitude (E)	Abbreviation
1	Sele River mouth—river water	40.2855	14.5633	Mouth
2	River mouth 500 m north—sea water	40.2904	14.5614	500N
3	River mouth 1000 m north—sea water	40.2912	14.5556	1000N
4	River mouth 1500 m north—sea water	40.2920	14.5538	1500N
5	River mouth 500 m west—sea water	40.2855	14.5612	500W
6	River mouth 1000 m west—sea water	40.2855	14.5550	1000W
7	River mouth 1500 m west—sea water	40.2855	14.5528	1500W
8	River mouth 500 m south—sea water	40.2847	14.5616	500S
9	River mouth 1000 m south—sea water	40.2839	14.5556	1000S
10	River mouth 1500 m south—sea water	40.2830	14.5538	1500S

**Table 2:** Pollution indices used in the study [44,48,49,50,51].

Index	Pollution Degree Criteria	Classification
I_geo_ (Geoaccumulation Index) [44]	I_geo_ < 0	Unpolluted
	0 < I_geo_ < 1	Unpolluted to moderately polluted
	1 < I_geo_ < 2	Moderately polluted
	2 < I_geo_ < 3	Moderately to heavily polluted
	3 < I_geo_ < 4	Heavily polluted
	4 < I_geo_ < 5	Heavily to extremely polluted
	I_geo_ > 5	Extremely polluted
CF (Contamination Factor) [50]	CF < 1	Low contamination
	1 ≤ CF <3	Moderate contamination
	3 ≤ CF < 6	Considerable contamination
	CF ≥ 6	Very high pollution
CD (Contamination Degree) [48]	CD < 6	Low contamination (Class 1)
	6 ≤ CD < 12	Moderate contamination (Class 2)
	12 ≤ CD < 24	Considerable contamination (Class 3)
	CD ≥ 24	Very high contamination (Class 4)
PLI (Pollution Load Index) [49]	PLI = 0	No pollution
	0 < PLI ≤ 1	Baseline levels of pollutants
	PLI > 1	Progressive deterioration of estuarine quality
PERI (Potential Ecological Risk Index) [51]	PERI < 150	Low pollution
	150 ≤ PERI < 300	Considerable/moderate pollution
	300 ≤ PERI ≤ 600	High/severe pollution
	PERI > 600	Very high/serious pollution

**Table 3:** Toxic response factor (T_r_^i^) of individual heavy metals [44,51].

Analyte	T_r_^i^
As	10 ^a,b^
Hg	40 ^c^
Cd	30 ^a^
Cr	2 ^a^
Cu	5 ^a,b^
Ni	5 ^a^
Pb	5 ^a,b^
Zn	1 ^a,b^

^a^ [44]; ^b^ [51]; ^c^ [52].

**Table 4:** Threshold effect level (TEL), probable effect level (PEL), effect range low (ERL) and effect range median (ERM) values for each heavy metal.

Heavy Metal	Unit	TEL ^a^	PEL ^a^	ERL ^b^	ERM ^b^
As	mg/kg dw	7.2	42	8.2	70
Hg	mg/kg dw	0.13	0.49	0.15	0.71
Cd	mg/kg dw	0.6	3.50	1.2	9.6
Cr	mg/kg dw	52.3	160.4	81.0	370
Cu	mg/kg dw	18.7	108.2	34.0	270
Ni	mg/kg dw	15.9	42.8	20.9	51.6
Pb	mg/kg dw	30.2	112.2	46.7	218
Zn	mg/kg dw	124	271	150	410

^a^ [54]; ^b^ [55].

**Table 5:** Parameters used for risk assessment due to ingestion and dermal exposure caused by heavy metals in water [61].

Parameter	Unit	Ingestion	Dermal Exposure
C_w_ (Heavy Metal Concentration)	µg/L	-	-
ADI (Average Daily Intake)	L/day	2.2	-
SA (Skin surface area)	cm^2^	-	18,000
K_p_ (Permeability coefficient)	cm/h	-	*
ET (Exposure Time)	h/event	-	0.58
EF (Exposure Frequency)	Days/years	365	350
EP (Exposure Duration)	Year	70	30
CF (Conversion Factor)	L/cm^3^	-	0.001
BW (Body Weight)	kg	70	70
ABS (Dermal Absorption Factor)	-	0.001	0.001
AT (Average Time)	Days	25,550	25,550

* K_p (Pb, Cu)_ = 0.001 cm/h, K_p (Cr)_ = 0.002 cm/h, K_p (Zn)_ = 0.006 cm/h, K_p (Ni)_ = 0.0002 cm/h [61]; K_p (As, Hg, Cd)_ = 0.001 cm/h [65,66].

**Table 6:** Reference oral doses (RfDs) (mg/kg/day) through oral ingestion and dermal contact by heavy metals in the water system.

HM	Unit	RfD_ingestion_	RfD_dermal_	Reference
As	mg/kg/day	0.30	-	[68]
Hg	mg/kg/day	n.a.	n.a.	[69]
Cd	mg/kg/day	0.50	0.005	[61]
Cr	mg/kg/day	3	0.015	[61]
Cu	mg/kg/day	40	12	[61]
Ni	mg/kg/day	20	5.4	[61]
Pb	mg/kg/day	1.4	0.42	[61]
Zn	mg/kg/day	300	60	[61]

n.a.: not available.

**Table 7:** Cancer slope factors (CSFs) (mg/kg/day) used for the carcinogenic risk assessment.

HM	Unit	CSF	Reference
As	mg/kg/day	1.50	[68]
Hg	mg/kg/day	n.a.	[69]
Cd	mg/kg/day	6.10	[61,68]
Cr	mg/kg/day	41.00	[61]
Cu	mg/kg/day	n.a.	[61,68]
Ni	mg/kg/day	0.84	[61,68]
Pb	mg/kg/day	8.50	[61,68]
Zn	mg/kg/day	n.a.	[61,68]

n.a.: not available.

**Table 8:** I_geo_ values for HMs detected in SED samples from the Sele River estuary, in southern Italy.

I_geo_ Value	As	Hg	Cd	Cr	Cu	Ni	Pb	Zn
I_geo_ max	0.744	3.389	1.900	−2.214	0.000	0.000	0.205	−1.763
I_geo_ min	−2.783	0.000	0.000	−6.790	−4.772	−5.250	−3.936	−4.589
I_geo_ average	−1.148	0.915	0.364	−4.418	−2.581	−2.104	−2.054	−3.321

**Table 9:** Pollution indices assessed for sediment (SED) samples collected during the sampling campaign near the Sele River estuary.

Pollution Index		As	Hg	Cd	Cr	Cu	Ni	Pb	Zn
CF		0.74	3.79	1.06	0.10	0.05	0.06	0.34	0.19
		Low	Considerable	Moderate	Low	Low	Low	Low	Low
E^i^_r_		7.35	151.43	31.80	0.20	0.25	0.31	1.68	0.19
Pollution index		Value	Classification						
CD		6.32	6 ≤ CD < 12 Moderate contamination (Class 2)						
PLI		0.30	0 < PLI ≤ 1 Baseline levels of pollutants						
PERI		193.21	150 ≤ PERI < 300 Considerable/moderate pollution						

**Table 10:** Heavy metal (HM) concentrations (µg/L) detected in dissolved phase (DP) samples collected from the study area with National Recommended Water Quality Criteria by the US EPA [58].

	As	Hg	Cd	Cr	Cu	Ni	Pb	Zn
CMC for Freshwater	340	1.4	1.8	586	/	470	65	120
CCC for Freshwater	150	0.77	0.72	85	/	52	2.5	120
% over CMC for Freshwater	0	60	0	0	/	0	0	0
% over CCC for Freshwater	0	18	27	0	/	0	14	0
CMC for Saltwater	69	1.8	33	1.1	4.8	74	210	90
CCC for Saltwater	36	0.94	7.9	50	3.1	8.2	8.1	81
% over CMC for Saltwater	0	6	0	58	10	0	0	0
% over CCC for Saltwater	0	19	0	0	31	13	6	0

CMC: Criterion Maximum Concentration (µg/L); CCC: Criterion Continuous Concentration (µg/L).

**Table 11:** Minimum, maximum, and mean values of CDIs (mg/kg/day) for HMs through different pathways.

HM	CDI_ingestion_			CDI_dermal_		
	Min	Max	Mean	Min	Max	Mean
As	2.17 × 10^−8^	4.11 × 10^−6^	6.63 × 10^−7^	4.23 × 10^−8^	8.02 × 10^−6^	1.29 × 10^−6^
Hg	1.26 × 10^−8^	3.74 × 10^−7^	7.30 × 10^−8^	2.45 × 10^−8^	7.29 × 10^−7^	1.42 × 10^−7^
Cd	1.60 × 10^−8^	1.97 × 10^−7^	5.16 × 10^−8^	3.13 × 10^−8^	3.84 × 10^−7^	1.01 × 10^−7^
Cr	2.36 × 10^−8^	1.44 × 10^−6^	2.44 × 10^−7^	9.19 × 10^−8^	5.62 × 10^−6^	9.53 × 10^−7^
Cu	5.00 × 10^−8^	4.81 × 10^−6^	9.32 × 10^−7^	9.75 × 10^−8^	9.38 × 10^−6^	1.77 × 10^−6^
Ni	5.63 × 10^−8^	3.06 × 10^−6^	5.60 × 10^−7^	2.19 × 10^−8^	1.19 × 10^−6^	2.19 × 10^−7^
Pb	5.28 × 10^−8^	3.18 × 10^−6^	6.90 × 10^−7^	1.03 × 10^−7^	6.19 × 10^−6^	1.38 × 10^−6^
Zn	6.82 × 10^−8^	3.48 × 10^−6^	8.11 × 10^−7^	7.98 × 10^−7^	4.08 × 10^−5^	9.49 × 10^−6^

**Table 12:** Minimum, maximum, and mean values of HIs evaluated for eight HMs (As, Hg, Cd, Cr, Cu, Ni, Pb, and Zn) through different pathways.

Value	HI_ingestion_	HI_dermal_	HI_total_
Min	9.11 × 10^−9^	6.60 × 10^−6^	2.68 × 10^−7^
Max	1.68 × 10^−5^	4.66 × 10^−4^	4.66 × 10^−4^
Mean	2.74 × 10^−6^	6.86 × 10^−5^	6.96 × 10^−5^

**Table 13:** The incremental lifetime cancer risk (ILCR) values obtained for total exposure due to both ingestion and dermal contact in the study area.

HM	ILCR		
	Min	Max	Mean
As	9.60 × 10^−8^	1.82 × 10^−5^	2.94 × 10^−6^
Cd	2.88 × 10^−7^	3.54 × 10^−6^	9.28 × 10^−7^
Cr	4.74 × 10^−6^	2.90 × 10^−4^	4.91 × 10^−5^
Ni	6.57 × 10^−8^	3.58 × 10^−6^	6.54 × 10^−7^
Pb	1.32 × 10^−6^	7.97 × 10^−5^	1.78 × 10^−5^
∑ILCR	6.51 × 10^−6^	3.95 × 10^−4^	7.14 × 10^−5^

## Data Availability

The data that support the findings of this study are available upon reasonable request from the corresponding author.

## Supplementary Materials

The following supporting information can be downloaded at: https://www.mdpi.com/article/10.3390/toxics12010038/s1, Table S1: Description of the sampling sites and heavy metals concentration (µg/L) detected in the water dissolved phase (DP) of the Sele River, southern Italy; Table S2: Description of the sampling sites and heavy metals concentration (µg/L) detected in Suspended Particulate Matter (SPM) of the Sele River, southern Italy; Table S3: Description of sampling sites and heavy metals concentration detected in April in the sediment samples (SED) (mg/kg dw) of Sele River, southern Italy; Table S4: Comparisons of heavy metal (HMs) concentrations detected in previous studies carried out from other river catchments and transitional waters in areas close to the study; Figure S1: PCA of the sediment data: Eigen values Plot (on the left) and PCA of the sediment data: Loading plot for the first and second principal component (on the right); Figure S2: PCA of the dissolved phase (DP) data: Eigen values Plot (on the left) and PCA of the sediment data: Loading plot for the first and second principal component (on the right); Figure S3: PCA of the suspended particulate matter (SPM) data: Eigen values Plot (on the left) and PCA of the SPM data: Loading plot for the first and second principal component (on the right).
