# Supplementary material for: Assessing Heavy Metals in the Sele River Estuary: An Overview of Pollution Indices in Southern Italy
Source: Toxics. 2024 Jan 3;12(1):38. doi: 10.3390/toxics12010038 (PMC10819315; doi:10.3390/toxics12010038)
Supplement: Supplementary file 1 [file toxics-12-00038-s001.zip › toxics-2772828-supplementary.pdf]

Assessing Heavy Metals in the Sele River Estuary: An Overview of Pollution Indices in Southern Italy

Fabiana Di Duca, Paolo Montuori *, Elvira De Rosa, Bruna De Simone, Immacolata Russo, Raffaele Nubi and Maria Triassi

Department of Public Health, University "Federico II", Via Sergio Pansini 5, 80131 Naples, Italy; fabianadiduca91@gmail.com (F.D.D.); derosaelvira92@gmail.com (E.D.R.); desimonebruna7@gmail.com (B.D.S.); imrusso@unina.it (I.R.); raf.nubi@gmail.com (R.N.); triassi@unina.it (M.T.)

* Correspondence: pmontuor@unina.it

Table S1. Description of the sampling sites and heavy metals concentration (μg/L) detected in the water dissolved phase (DP) of the Sele River, southern Italy.

Location ID	Season	As	Hg	Cd	Cr	Cu	Ni	Pb	Zn
1 (river water)	July	6.84 ± 3.15	2.35 ± 1.13	1.45 ± 0.48	14.97 ± 4.97	3.02 ± 1.00	11.15 ± 3.73	14.41 ± 4.78	4.17 ± 1.37
	November	1.17 ± 0.51	0.23 ± 0.09	0.52 ± 0.18	3.95 ± 1.32	< 1.57	1.94 ± 0.63	1.64 ± 0.53	2.25 ± 0.77
	February	1.55 ± 0.73	< 0.23	< 0.50	0.91 ± 0.32	< 1.57	< 1.73	< 1.57	2.18 ± 0.71
	April	3.32 ± 1.60	0.57 ± 0.18	0.63 ± 0.21	11.12 ± 3.69	1.67 ± 0.54	5.58 ± 1.84	4.82 ± 1.62	2.69 ± 0.87
2 (sea water)	July	5.70 ± 1.87	0.72 ± 0.22	0.84 ± 0.29	3.71 ± 1.18	1.79 ± 1.27	2.81 ± 0.94	1.59 ± 0.52	5.46 ± 1.83
	November	0.69 ± 0.22	< 0.23	< 0.50	0.62 ± 0.17	< 1.57	< 1.73	< 1.57	< 2.17
	February	< 0.67	< 0.23	< 0.50	< 0.63	< 1.57	< 1.73	< 1.57	< 2.17
	April	2.41 ± 0.81	< 0.23	< 0.50	1.96 ± 0.66	1.58 ± 0.54	2.12 ± 0.69	< 1.57	2.92 ± 0.97
3 (sea water)	July	2.58 ± 0.85	0.25 ± 0.09	0.53 ± 0.18	1.54 ± 0.52	1.71 ± 0.56	1.76 ± 0.61	< 1.57	2.21 ± 0.75
	November	< 0.67	< 0.23	< 0.50	0.64 ± 0.19	< 1.57	< 1.73	< 1.57	< 2.17
	February	< 0.67	< 0.23	< 0.50	< 0.63	< 1.57	< 1.73	< 1.57	< 2.17
	April	0.74 ± 0.25	< 0.23	< 0.50	< 0.63	1.57 ± 0.52	< 1.73	< 1.57	< 2.17
4 (sea water)	July	0.68 ± 0.28	< 0.23	< 0.50	0.68 ± 0.20	< 1.57	< 1.73	< 1.57	< 2.17
	November	< 0.67	< 0.23	< 0.50	< 0.63	< 1.57	< 1.73	< 1.57	< 2.17
	February	< 0.67	< 0.23	< 0.50	< 0.63	< 1.57	< 1.73	< 1.57	< 2.17
	April	< 0.67	< 0.23	< 0.50	< 0.63	< 1.57	< 1.73	< 1.57	< 2.17
5 (sea water)	July	2.36 ± 0.79	0.92 ± 0.28	0.82 ± 0.24	6.17 ± 2.04	1.66 ± 0.53	5.81 ± 1.91	4.43 ± 1.45	2.25 ± 0.72
	November	< 0.67	< 0.23	< 0.50	1.65 ± 0.52	< 1.57	< 1.73	< 1.57	< 2.17
	February	0.52 ± 0.16	< 0.23	< 0.50	< 0.63	< 1.57	< 1.73	< 1.57	< 2.17
	April	1.39 ± 0.44	0.23 ± 0.05	0.52 ± 0.16	5.36 ± 1.74	1.59 ± 0.49	2.34 ± 0.75	1.90 ± 0.61	< 2.17
6 (sea water)	July	< 0.67	0.42 ± 1.21	0.60 ± 0.19	1.93 ± 0.61	1.57± 0.51	3.58 ± 1.15	1.68 ± 0.53	< 2.17
	November	< 0.67	< 0.23	< 0.50	0.65 ± 0.19	< 1.57	< 1.73	< 1.57	< 2.17
	February	< 0.67	< 0.23	< 0.50	< 0.63	< 1.57	< 1.73	< 1.57	< 2.17
	April	< 0.67	< 0.23	< 0.50	3.50 ± 1.13	< 1.57	1.85 ± 0.57	< 1.57	< 2.17
7 (sea water)	July	< 0.67	< 0.23	< 0.50	0.79 ± 0.23	< 1.57	1.76 ± 0.56	< 1.57	< 2.17
	November	< 0.67	< 0.23	< 0.50	< 0.63	< 1.57	< 1.73	< 1.57	< 2.17
	February	< 0.67	< 0.23	< 0.50	< 0.63	< 1.57	< 1.73	< 1.57	< 2.17

	April	< 0.67	< 0.23	< 0.50	1.01 ± 0.31	< 1.57	< 1.73	< 1.57	< 2.17
8 (sea water)	July	14.28 ± 4.73	1.87 ± 0.57	1.69 ± 0.52	12.41 ± 4.09	13.23 ± 4.42	8.73 ± 2.88	3.12 ± 1.00	3.32 ± 1.09
	November	1.15 ± 0.36	0.25 ± 0.07	0.54 ± 0.14	2.89 ± 0.93	3.74 ± 1.20	1.84 ± 0.60	< 1.57	2.19 ± 0.71
	February	6.47 ± 2.10	< 0.23	< 0.50	0.68 ± 0.18	< 1.57	< 1.73	< 1.57	< 2.17
	April	1.62 ± 0.53	0.61 ± 0.19	0.82 ± 0.23	11.54 ± 3.81	7.52 ± 2.48	3.62 ± 1.17	1.60 ± 0.51	2.35 ± 0.75
9 (sea water)	July	7.59 ± 2.49	1.45 ± 0.45	0.73 ± 0.21	5.72 ± 1.88	5.42 ± 1.78	3.79 ± 1.22	< 1.57	2.38 ± 0.71
	November	< 0.67	< 0.23	< 0.50	1.05 ± 0.33	< 1.57	< 1.73	< 1.57	< 2.17
	February	1.13 ± 0.31	< 0.23	< 0.50	0.67 ± 0.19	< 1.57	< 1.73	< 1.57	< 2.17
	April	2.91 ± 0.92	0.34 ± 0.09	0.50 ± 0.13	4.48 ± 1.41	3.56 ± 1.11	1.89 ± 0.59	< 1.57	2.18 ± 0.65
10 (sea water)	July	3.30 ± 0.97	0.42 ± 0.11	0.58 ± 0.15	2.69 ± 0.85	2.35 ± 0.73	1.80 ± 0.56	< 1.57	2.20 ± 0.72
	November	< 0.67	< 0.23	< 0.50	0.71 ± 0.21	< 1.57	< 1.73	< 1.57	< 2.17
	February	< 0.67	< 0.23	< 0.50	< 0.63	< 1.57	< 1.73	< 1.57	< 2.17
	April	1.21 ± 0.37	< 0.23	0.51 ± 0.13	1.30 ± 0.39	1.58 ± 0.48	< 1.73	< 1.57	2.17 ± 0.66

Table S2. Description of the sampling sites and heavy metals concentration (μg/L) detected in Suspended Particulate Matter (SPM) of the Sele River, southern Italy.

Location ID	Season	As	Hg	Cd	Cr	Cu	Ni	Pb	Zn
1 (river water)	July	75.23 ± 24.97	6.94 ± 2.29	2.06 ± 0.61	17.45 ± 5.73	100.69 ± 33.41	54.03 ± 17.78	86.66 ± 28.73	90.28 ± 29.95
	November	19.65 ± 6.45	2.11 ± 0.64	0.52 ± 0.15	3.20 ± 1.02	18.54 ± 6.08	10.34 ± 3.38	33.28 ± 11.01	2.19 ± 0.67
	February	4.59 ± 1.52	1.84 ± 0.51	< 0.50	1.54 ± 0.47	8.96 ± 2.85	2.85 ± 0.85	13.50 ± 4.44	< 2.17
	April	37.82 ± 12.53	2.47 ± 0.76	1.01 ± 0.30	7.43 ± 2.38	71.21 ± 23.64	24.86 ± 8.19	53.07 ± 17.67	30.24 ± 10.03
2 (sea water)	July	20.31 ± 6.65	3.07 ± 1.00	0.74 ± 0.22	9.76 ± 3.18	36.77 ± 12.16	7.02 ± 2.27	19.35 ± 6.32	29.05 ± 9.61
	November	5.24 ± 1.72	0.48 ± 0.13	< 0.50	2.07 ± 0.66	11.52 ± 3.81	5.93 ±1.85	10.47 ± 3.40	2.19 ± 0.63
	February	8.50 ± 2.77	0.40 ± 0.09	< 0.50	1.58 ± 0.47	1.88 ± 0.53	< 1.73	1.68 ± 0.53	< 2.17
	April	0.85 ± 0.23	1.05 ± 0.32	< 0.50	4.27 ± 1.33	25.50 ± 8.41	8.96 ± 2.88	22.81 ± 7.46	14.78 ± 4.81
3 (sea water)	July	7.42 ± 2.44	1.86 ± 0.60	< 0.50	4.91 ± 1.58	4.63 ± 1.44	4.21 ± 1.31	8.29 ± 2.65	10.39 ± 3.34
	November	2.54 ± 0.82	0.69 ±0.18	< 0.50	1.01 ± 0.31	17.82 ± 5.84	< 1.73	4.54 ± 1.43	< 2.17
	February	< 0.67	< 0.23	< 0.50	0.89 ± 0.25	< 1.57	< 1.73	< 1.57	< 2.17
	April	6.87 ± 2.25	0.54 ± 0.16	< 0.50	1.67 ± 0.49	9.58 ± 3.08	5.08 ± 1.58	10.32 ± 3.32	6.59 ± 2.08
4 (sea water)	July	0.75 ± 0.24	0.78 ± 0.24	0.51 ± 0.15	2.64 ± 0.77	< 1.57	< 1.73	2.21 ± 0.65	5.07 ± 1.58
	November	0.69 ± 0.19	0.63 ± 0.17	< 0.50	1.36 ± 0.35	8.96 ± 2.89	< 1.73	< 1.57	< 2.17
	February	< 0.67	< 0.23	< 0.50	0.87 ± 0.23	< 1.57	< 1.73	< 1.57	< 2.17
	April	1.29 ± 0.42	< 0.23	< 0.50	0.75 ± 0.19	4.33 ± 1.31	1.79 ± 0.52	3.86 ± 1.21	2.17 ± 0.59
5 (sea water)	July	42.08 ± 13.99	4.97 ± 1.63	1.03 ± 0.25	10.10 ± 3.29	64.91 ± 21.53	21.05 ± 6.92	53.69 ± 17.81	47.88 ± 15.86
	November	12.69 ± 4.2	1.05 ± 0.29	< 0.50	2.62 ± 0.82	25.83 ± 8.51	11.27 ± 3.64	24.78 ± 8.15	< 2.17
	February	1.96 ± 0.63	0.91 ± 0.24	< 0.50	1.87 ± 0.58	5.17 ± 1.62	2.18 ± 0.66	5.21 ± 1.65	< 2.17
	April	18.37 ± 6.08	1.56 ± 0.48	0.65 ± 0.12	5.28 ± 1.73	41.87 ± 12.95	21.29 ± 6.08	34.65 ± 11.47	20.67 ± 6.78
6 (sea water)	July	28.93 ± 9.61	3.21 ± 1.03	0.87 ± 0.21	7.41 ± 2.44	26.53 ± 8.75	10.40 ± 3.36	30.20 ± 9.94	24.89 ± 8.21
	November	5.04 ± 1.66	1.10 ± 0.32	< 0.50	1.09 ± 0.32	9.65 ± 3.10	3.25 ± 1.01	8.57 ± 2.77	< 2.17
	February	0.78 ± 0.24	0.74 ± 0.21	< 0.50	0.88 ± 0.24	1.59 ± 0.45	< 1.73	1.84 ± 0.53	< 2.17
	April	8.65 ± 2.78	0.86 ± 0.27	0.59 ± 0.21	2.17 ± 0.66	26.84 ± 8.86	12.36 ± 4.03	19.86 ± 6.52	11.56 ± 3.74
7 (sea water)	July	12.09 ± 3.97	1.23 ± 0.36	< 0.50	3.13 ± 1.01	6.21 ± 2.00	6.18 ± 1.95	9.28 ± 2.96	9.21 ± 2.96
	November	1.84 ± 0.55	0.87 ± 0.25	< 0.50	1.05 ± 0.28	4.07 ± 1.25	< 1.73	2.70 ± 0.83	< 2.17
	February	< 0.67	< 0.23	< 0.50	< 0.63	< 1.57	< 1.73	< 1.57	< 2.17

	April	2.07 ± 0.63	0.52 ± 0.12	0.54 ± 0.09	1.21 ± 0.37	8.03 ± 2.56	3.16 ± 0.94	8.95 ± 2.77	4.58 ± 1.45
8 (sea water)	July	116.52 ± 38.52	10.03 ± 3.27	4.57 ± 1.49	33.47 ± 11.06	139.85 ± 46.51	88.74 ± 29.47	81.92 ± 27.12	107.54 ± 35.68
	November	30.26 ± 9.86	3.11 ± 0.95	0.71 ± 0.18	8.56 ± 2.78	39.57 ± 13.08	21.06 ± 6.93	39.87 ± 13.08	2.25 ± 0.63
	February	12.71 ± 4.18	1.07 ± 0.33	< 0.50	4.03 ± 1.26	18.61 ± 6.09	3.29 ± 1.02	13.66 ± 4.41	< 2.17
	April	56.88 ± 18.72	3.95 ± 1.24	2.10 ± 0.62	15.69 ± 5.19	92.05 ± 30.56	31.68 ± 10.44	59.40 ± 19.69	42.79 ± 14.13
9 (sea water)	July	72.00 ± 23.78	4.20 ± 1.38	1.34 ± 0.35	14.28 ± 4.67	58.96 ± 19.53	44.52 ± 14.73	30.07 ± 9.92	48.96 ± 16.22
	November	19.65 ± 6.43	1.08 ± 0.35	0.52 ± 0.12	3.17 ± 0.96	23.44 ± 7.70	10.37 ± 3.35	18.92 ± 6.21	< 2.17
	February	4.21 ±1.34	0.79 ± 0.22	< 0.50	1.29 ± 0.37	6.72 ± 2.13	1.87 ± 0.51	5.21 ± 1.65	< 2.17
	April	31.52 ± 10.46	2.04 ± 0.61	0.93 ± 0.26	6.89 ± 2.15	39.57 ± 13.07	20.25 ± 6.62	28.54 ± 9.43	23.63 ± 7.76
10 (sea water)	July	24.19 ± 7.95	2.48 ± 0.80	0.69 ± 0.19	6.08 ± 1.89	23.91 ± 7.86	19.88 ± 6.54	8.88 ± 2.85	27.30 ± 8.98
	November	6.30 ± 2.07	0.86 ± 0.26	< 0.50	1.92 ± 0.54	11.69 ± 3.82	4.19 ±1.33	6.23 ± 1.78	< 2.17
	February	1.04 ± 0.33	< 0.23	< 0.50	0.75 ± 0.17	2.05 ± 0.59	< 1.73	3.17 ± 0.97	< 2.17
	April	9.89 ± 3.23	1.17 ± 0.31	0.53 ± 0.17	3.45 ± 1.08	16.80 ± 5.47	10.34 ± 3.31	11.95 ± 3.84	13.92 ± 6.59

Table S3. Description of sampling sites and heavy metals concentration detected in April in the sediment samples (SED) (mg/kg dw) of Sele River, southern Italy.

Location ID	As	Hg	Cd	Cr	Cu	Ni	Pb	Zn
1 (river water)	5.96 ± 2.84	0.74 ± 0.33	0.28 ± 0.12	20.35 ± 10.16	7.32 ± 3.54	9.27 ± 4.55	10.63 ± 5.27	33.64 ± 16.72
2 (sea water)	3.21 ± 1.51	< 0.70	< 0.25	5.28 ± 2.63	2.96 ± 1.31	2.68 ± 1.30	3.42 ± 1.57	14.05 ± 6.88
3 (sea water)	1.09 ± 0.49	< 0.70	< 0.25	2.64 ± 1.27	2.47 ± 1.21	< 2.67	2.05 ± 0.96	8.64 ± 4.09
4 (sea water)	< 1.07	< 0.70	< 0.25	1.22 ± 0.56	< 2.47	< 2.67	< 1.87	6.21 ± 2.97
5 (sea water)	4.12 ± 2.03	< 0.70	< 0.25	10.37 ± 5.13	3.64 ± 1.73	3.02 ± 1.42	6.28 ± 3.12	19.93 ± 9.84
6 (sea water)	2.05 ± 0.98	< 0.70	< 0.25	4.28 ± 1.99	< 2.47	< 2.67	1.96 ± 0.89	9.47 ± 4.23
7 (sea water)	1.93 ± 0.95	< 0.70	< 0.25	5.02 ± 2.48	< 2.47	< 2.67	< 1.87	5.92 ± 2.87
8 (sea water)	12.56 ± 6.12	1.10 ± 0.51	0.25± 0.11	29.10 ± 14.45	3.18 ± 1.53	24.89 ± 12.34	34.57 ± 17.07	42.00 ± 20.82
9 (sea water)	4.47 ± 2.19	0.81 ± 0.38	< 0.25	8.95 ± 4.42	2.59 ± 1.19	2.75 ± 1.35	6.02 ± 2.67	24.27 ± 12.05
10 (sea water)	1.38 ± 0.62	< 0.70	< 0.25	5.03 ± 2.45	< 2.47	nd	2.26 ± 1.01	12.06 ± 5.91

Table S4. Comparisons of heavy metal (HMs) concentrations detected in previous studies carried out from other river catchments and transitional waters in areas close to the study.

Area	References	Heavy Metals							
		**As**	**Hg**	**Cd**	**Cr**	**Cu**	**Ni**	**Pb**	**Zn**
**DPs (μg/L^{-1})**									
Sarno River, Italy	Montuori et al., [72]	3.10 - 28.57	0.1 – 0.74	0.03 – 0.79	41.63- 1669.84	0.11 – 9.51	0.47 – 22.11	0.41 – 10.47	0.14 – 5.17
Tiber River, Italy	Montuori et al., [26]	2.05 - 92.04	0.1 – 0.90	0.06 – 5.42	1.05 – 94.61	0.34 – 821.12	0.33 – 77.92	1.08 – 186.74	0.16 – 155.38
Volturno River, Italy	De Rosa et al., [35]	0.52 – 15.07	0.10 – 2.72	0.09 – 3.00	1.00 – 15.77	0.10 – 14.00	0.10 – 11.75	0.45 – 15.22	0.11 – 6.20
This study	Di Duca et al.,	nd – 14.28	nd – 2.35	nd – 1.69	nd – 14.97	nd – 13.23	nd – 11.15	nd – 14.41	nd – 5.46
**SPM (μg/L^{-1})**									
Sarno River, Italy	Montuori et al., [72]	1.94 – 106.7	0.06 – 134.2	0.03 – 0.62	14.1 – 1149.7	36.3 – 809.8	3.3 – 1009.3	1.02 – 4063.5	59.4 – 3327.2
Tiber River, Italy	Montuori et al., [26]	2.01 – 243.9	0.11 – 6.37	0.05 – 4.80	1.02 – 131.6	5.21 – 1550.3	0.68 – 172.5	0.62 – 272.9	0.32 – 505.2
Volturno River, Italy	De Rosa et al., [35]	1.00 – 120.3	1.01 – 12.07	0.01 – 5.89	1.10 – 35.55	0.63 – 142.65	0.52 – 91.27	1.00 – 89.67	0.01 – 110.12
This study	Di Duca et al.,	nd – 116.52	nd – 10.03	nd – 4.57	nd – 33.47	nd – 139.85	nd – 88.74	nd – 86.66	nd – 107.54
**SED (μg/kg^{-1})**									
Sarno River, Italy	Montuori et al., [72]	0.24 – 69.3	0.2 – 1.02	0.39 – 2.92	23.77 – 514.40	33.64 – 580.18	1.58 – 651.70	0.47 – 1658.1	55.00 – 802.8
Tiber River, Italy	Montuori et al., [26]	2.65 – 45.2	0.05 – 1.14	0.08 – 2.22	8.52 – 189.91	16.01 – 527.81	6.22 – 494.26	3.07 – 186.67	6.18 – 413.36
Volturno River, Italy	De Rosa et al., [35]	1.00 – 14.08	0.06 – 1.45	0.10 – 0.32	2.92 – 31.10	1.67 – 8.40	0.88 – 26.49	0.65 – 36.27	7.03 – 43.06
This study	Di Duca et al.	nd – 12.56	nd – 1.10	nd – 0.98	1.22 – 29.10	nd – 7.32	nd – 24.89	nd – 34.57	5.92 – 42.00

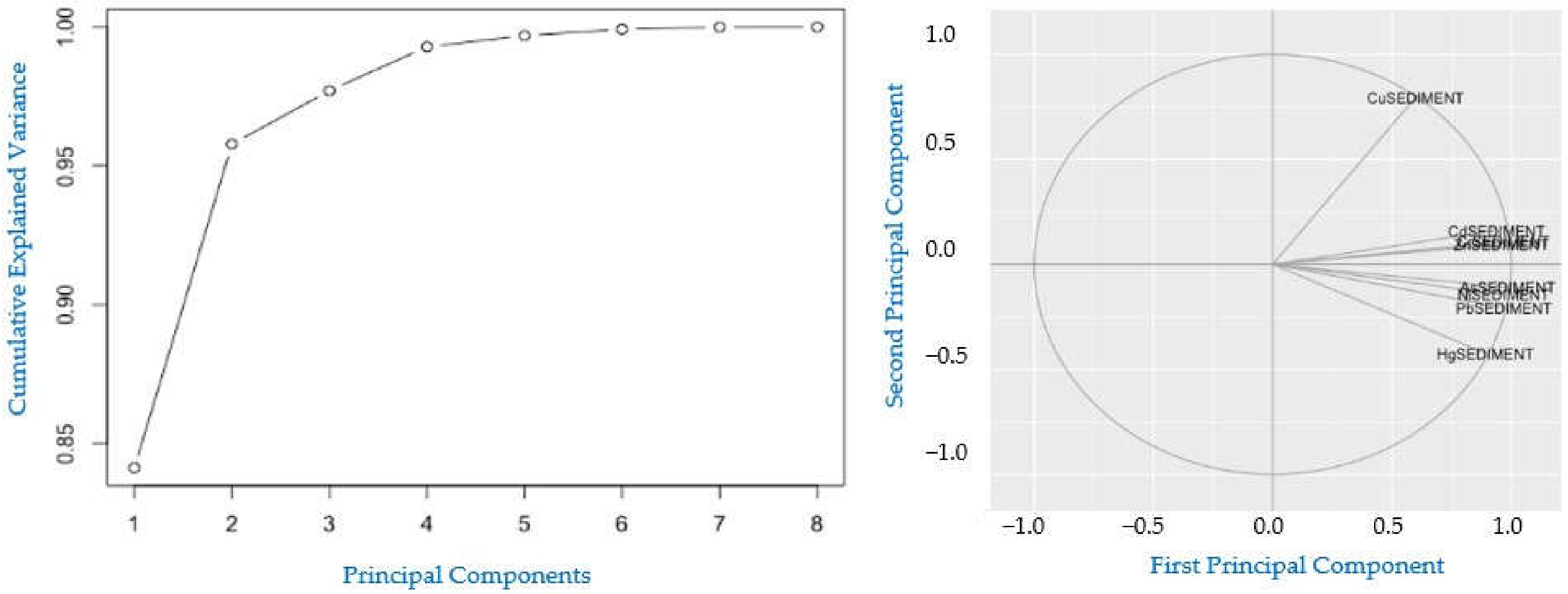

Figure S1. PCA of the sediment data: Eigen values Plot (on the left) and PCA of the sediment data: Loading plot for the first and second principal component (on the right).

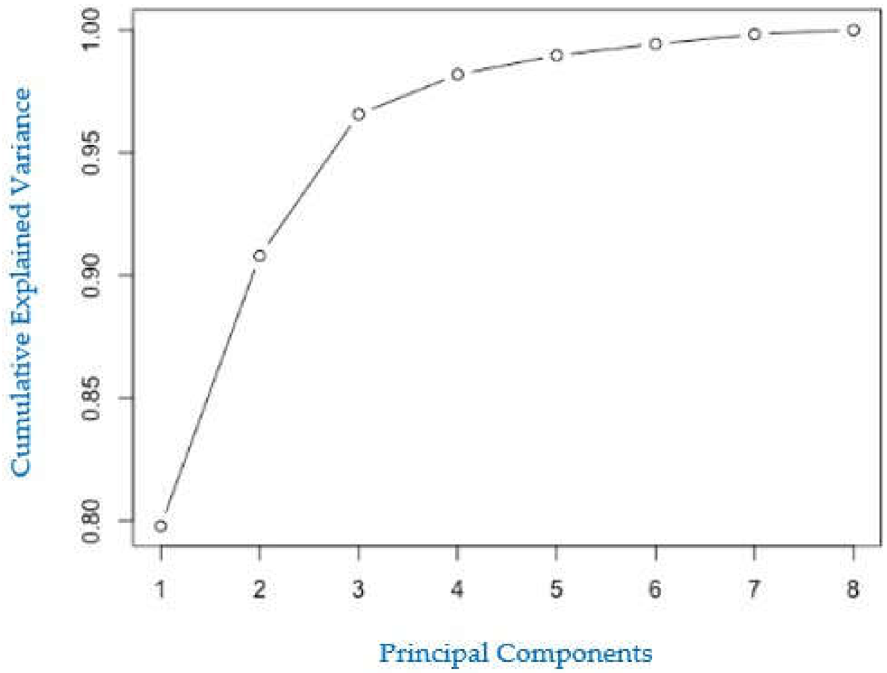

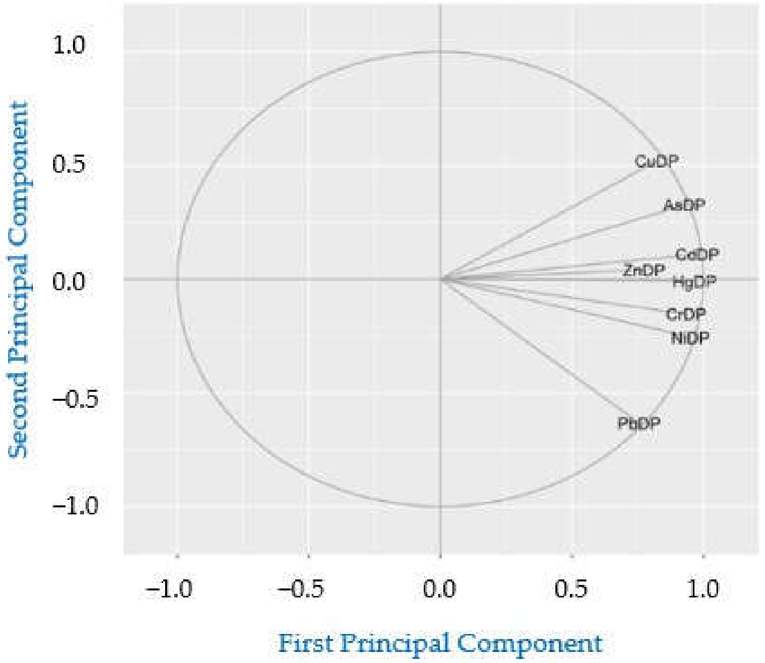

Figure S2. PCA of the dissolved phase (DP) data: Eigen values Plot (on the left) and PCA of the sediment data: Loading plot for the first and second principal component (on the right).

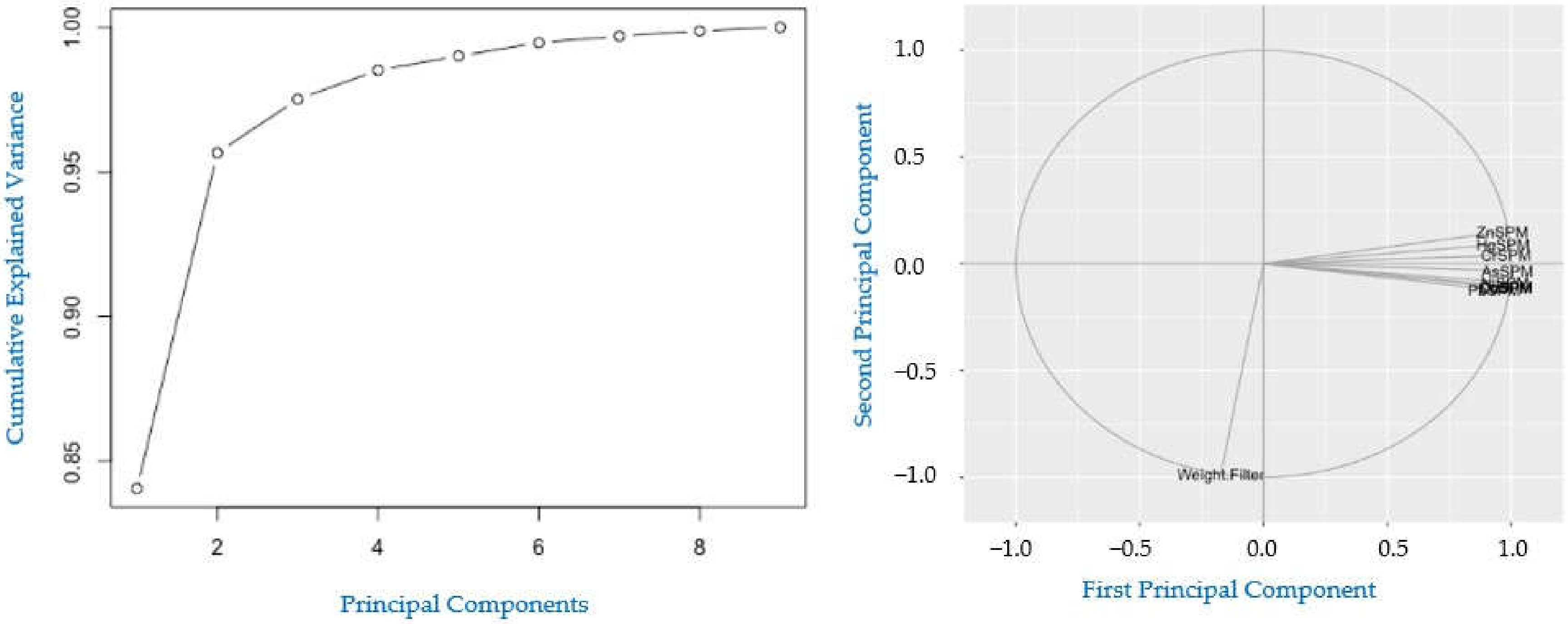

Figure S3. PCA of the suspended particulate matter (SPM) data: Eigen values Plot (on the left) and PCA of the SPM data: Loading plot for the first and second principal component (on the right).
